# Big data analyses for real-time tracking of risks in the mineral raw material markets: implications for improved supply chain risk management

**DOI:** 10.1007/s13563-022-00337-z

**Published:** 2022-09-12

**Authors:** Peter Buchholz, Arne Schumacher, Siyamend Al Barazi

**Affiliations:** German Mineral Resources Agency (DERA) at the Federal Institute for Geosciences and Natural Resources (BGR), Wilhelmstrasse 25-30, 13593 Berlin, Germany

**Keywords:** Big data, Supply chain, Market

## Abstract

Resilient supply chains have become a key issue for manufacturing companies to ensure a stable supply for their manufacturing processes and for governments to ensure the stable supply of essential goods to society. Building diversified supply chains and monitoring the performance of suppliers are major mitigation strategies to counteract disruptions at an early stage. Supply chain risk management and monitoring of supply chains using big data analytics are getting increasing attention. The growing number of data sources has huge implications on the reporting of incidents that may disrupt supply chains. The data sources may stem from a variety of internet sources like daily media reports provided on websites, social media or specialist media, or they may stem from specific databases. The sooner this information is disclosed to stakeholders and analysed the better the preventive strategies generally are. Timely information prolongs the reaction time and may help to reduce the severity of an incident. This paper highlights a science-based real-time tracking analysis of risks in the mineral raw material markets for the period 2019 to 2021 using big data analytics provided by a commercial system. A set of data for 12 selected mineral raw materials was provided by the authors and analysed using more than 100 risk indicators from 14 major risk categories as part of a commercial big data system. The extracted information can have imminent value to identify supply shortages, production halts or price peaks at an early stage. The main question was to find out whether such big data analytics are precise enough to detect potential, globally relevant, supply shortages in mineral raw material markets in due time. The results of this paper show that using big data analytics can be a very effective tool to extract relevant information about supply sources and to react timely in case of disruptions or social or environmental mismanagement on the supplier side. However, the nature of big data sources suggests that the parameters of the applied models need elaborate configuration. Each raw mineral market has its own peculiarities in terms of volume, mode of transport, market concentration or countries of origin. These factors influence the relevance of the reported incidents. Furthermore, some incidents have a spurious or only minor connection to the individual markets. For these reasons, we conclude that only supervised models reap the most benefits in the monitoring of mineral raw material markets.

**Supplementary Information:**

The online version contains supplementary material available at 10.1007/s13563-022-00337-z.

## Introduction

The past 3 years have shown a dramatic increase in price and supply risks and high price volatility, driven by (i) the USA-China trade war which escalated in 2020, (ii) the COVID-19 pandemic starting 2020, (iii) climate political measures in China combined with an economic transformation and (iv) the Russian invasion into Ukraine in February 2022 followed by economic sanctions against the Russian Federation. The interlocking developments have impacted on global production, investment into new production capacities, global trade including transport logistics, as well as global financial stability with respect to increasing national depts, prolongation of low interest rates and rising inflation. On top of that, national infrastructure programs, the global energy transition and new mobility concepts are driving the demand for energy and mineral raw materials causing pressure to supply of the raw materials. Resilient supply chains have become a key issue for manufacturing companies to ensure a stable supply for their manufacturing processes and for governments to ensure the stable supply of essential goods to society. Our study intends to push forward a discussion to improve the resilience of supply chains by using big data analytics applied to mineral raw material markets.

This study highlights the results of big data analyses applied to the mineral raw material markets, particularly in the mining sector, which stands at the beginning of many supply chains. Timely information is a key influence for performance of companies, competitiveness, sustainability, eco-friendliness and customer satisfaction. It also is a source of various types of incidents that may affect supply chains. These incidents are unforeseen or predictive, exogenous or endogenous in nature. Common to all these types is the importance to acknowledge the incidents in due time in order to devise prudent management decisions to mitigate adverse effects.

The major research questions are as follows: (i) whether such big data analyses and combined alerting systems are precise enough to detect potential, globally relevant, supply shortages for individual mineral raw material markets in due time; (ii) which risk indicators are most relevant for mineral raw material markets and how they differ between these markets; and (iii) how to improve the relevance of results to better apply big data analyses to mineral raw material markets. To our knowledge, big data analyses for real-time tracking of supply risks applied to mineral raw material markets have not yet been scientifically analysed and evaluated in such detail. Commercial supply chain management tools do exist since about 10 years time, are expensive and were originally programmed to monitor the performance of specific suppliers along a companies’ supply chain. For this study, we designed a global approach to specifically analyse global mineral raw material markets as a whole. The approach is rather simple: We selected the largest mines for several mineral raw material markets, calculated their share of global production and studied—a period of 1.5 years—to see how certain risk incidents disrupted important mines or supply routes and how they influenced the global market. There is a big research gap in ranking the severity of risks and how they might be classified more correctly with computer intelligence. The current approach cannot answer all these questions but can be a starting point for further research.

The paper is structured as follows:

- Section 2 gives a background about fundamental terminology and methodology with respect to big data analytics and tools and data sources which were used for this study. The data sources vary greatly in form and detail and may include economic, environmental, political, social, regulatory or operational risks affecting supply. Since the variety and volume of data sources are constantly increasing, big data and machine learning techniques also grow more important. Section 2 hence also gives some background about big data and machine learning techniques, which are profoundly useful for real-time tracking of incidents in mineral raw material markets.
- Section 3 briefly reviews the methodology of a commercial supply chain risk management tool, provided by Everstream Analytics (2021). The tool deploys big data analytics to turn data into structured information for supply chain risk analysis. The information age produces a huge amount of data that may be used for analyses once adequately processed. The aim of such information systems is to inform decision-makers in industry and politics more timely about risks affecting supply chains and in this case mineral raw materials markets. The development of such information systems incorporates various data sources and incidents affecting global production and distribution of mineral raw materials. The benefits of supplying intelligence faster and more accurately not only improve the basis for decision-making but also extend the reaction time for disruptions. Section 3 also presents an adapted approach for this study developed by the authors and outlines the structure of the analysis in detail.
- Section 4 presents the results of the monitoring and real-time tracking analysis implementing a big data and machine learning framework provided by the Everstream Analytics tool (former DHL Resilience360). This section also highlights examples of the techniques that are applied to set up an early warning system for raw materials markets and to put forward examples that affected these markets, most often detrimentally. For the period 2019 to 2021, 12 mineral raw materials and relevant mine sites and company headquarters were tracked for a wide range of incidents and events. Out of these 12 mineral raw materials, five were studied in more detail, and the exemplary results of the tracking analysis are presented.
- Section 5 discusses the results and the applicability and capability of such big data applications in relation to mineral raw material markets. We discuss many examples of detected risks incidents, which had the potential to severely affect individual supply chains, whereby others were irrelevant (natural occurrence risks, transportation risks, socio-political risks, supplier risks). We also calculate how much of global production was at risk for a single global mineral raw material market. The discussion closes with some remarks about limitations of big data analytics and sources and gives a brief outlook about further applications of big data analytics in mineral raw material markets.
- Section 6 presents the conclusions and points to further research.

## Background

### Supply chain management challenges

The quest for improving production and supply chain management is a perpetual endeavour. For one, whatever brings down costs is not only added value for the economy and the consumer but also a desirable competitive advantage. Just-in-time (JIT) and make to order (MTO) are only possible and will only be successful if time and resources are reduced all throughout the supply chain. Information can improve virtually all processes in the economy, from manufacturing to distribution, warehousing, demand forecasting, pricing, product quality and safety, customer tastes and expectations and supplier evaluation or selection. Minimising inventories and production cycle times along the supply chain may not only be a significant money saver and reduce customer delivery lead times; it also consumes less resources and thereby protects environmental exploitation. Further, social diligence ensures increased transparency, traceability and security in the supply of goods. Today, numerous efforts, such as the American Dodd-Frank Act (U.S. Congress 2022), the European counterpart directive 2017/21 or the Organisation for Economic Cooperation and Development (OECD) “Due Diligence Guidance for Responsible Supply Chains of Minerals from Conflict-Affected and High-Risk Areas” (OECD 2022) as well as advancing national initiatives, e.g. Germany’s supply chain due diligence law (Bundesministerium für wirtschaftliche Zusammenarbeit und Entwicklung, 2022), increase the need for enterprises to take on responsibility how their goods are produced and shipped in terms of their environmental and social costs. These regulatory efforts are increasingly expected by customers that want their products to be manufactured sustainably and responsibly. Especially in the trade of primary resources, these materials are often found and extracted in volatile and unstable countries with adverse consequences for the labour force and environment. Additionally, more transparency and information alleviate mitigation measures in case of disruptions or supply shortages. Enterprises with more information will find it easier to improve their decision-making processes by finding quick alternatives that ultimately increase the resilience of the supply chain. Rising consumer expectations and competition also increase the need to shorten the production cycle and supply goods that are uninterruptedly and persistently available. It is hence a logical strive to improve the efficiency of these processes. Today’s supply chains use machine learning algorithms and big data sources to anticipate and/or react to changes in the supply chain and for a leaner order, production and inventory management. Though these measures may seem to have high initial investment costs, the long-term benefits will increase the quality of responses in the business cycle and strengthen efforts to counteract inevitable disruptions.

### Types of supply chain risks and mitigation of risks

Supply chains today are highly globalised, especially so in the mineral raw material markets. This is partly due to the fact that geological mineral reserves are spread out to each continent and are mined according to their economic value in the global markets. Secondly, there is a growing trend for companies to focus on their core competences resulting in a fragmentation of the supply chain. This trend leads to the outsourcing of distribution and transportation networks with less control, more interdependencies and a growing number of actors in the supply chain (Kersten et al. 2015). Thirdly, as competitive countries engage in trade, they seek favourable factors of production. In general, this particularly applies to the mineral raw materials markets in terms of refining, and they settle for locations where auspicious conditions are found, one example being cheap(er) labour. Since these outweigh transportation costs, longer transportation networks are accepted. These factors contribute to expanded transportation routes with more players in the supply chain. For these reasons, global networks make it harder to control the entire supply chain. There might be a loss of control and visibility along the supply chain the further one goes down the tiers. In such a fragmented market, even seemingly small incidents may cause a major disruption in world trade. The 2021 Suez Canal obstruction wedged the vessel Ever Given sideways in the canal and blocked it for 6 days. This prevented an estimated $9.6 billion in world trade (nbc News, 2021a, b). The recent COVID-19 pandemic also exposed the vulnerability of today’s global supply chains to such an extent that supermarket and electronic store shelves were empty with products even in the most developed countries due to port closures in Yantian, China, the 4th largest container vessel port in the world, in relation with a COVID-19 outbreak in June 2021. This incident had even more far-reaching consequences for the global trade and delayed the shipments of 160,000 40-feet containers. In its aftermath, worldwide product and transportation prices increased, and deliveries were delayed (Frankfurter Rundschau 2021).

The incidents may also be very different in nature, ranging from weather, social, regulatory to economic incidents. The nature of incidents—and with it the processing of incoming information—can be either predictive or unforeseen. The former group of incidents is by and large plannable through good and lean management decisions. The latter type of incidents—unforeseen—cannot as easily be proactively managed and is reactive in nature. Mostly, these are exogenous to the supply chain, and examples are environmental and social disruptions.

For these reasons, the terminology used in supply chain risk management (SCRM) unfolds positive as well as negative connotations. Among the positive vocabulary are robustness, resilience, agility, flexibility and transparency. The negative terms often referred to are vulnerability, disturbance, disruption, uncertainty, crisis, disaster, threat etc. Baryannis et al. (2019) provide a comprehensive overview of their differences and purport the following SCRM definition:

Supply chain risk management encompasses the collaborative and coordinated efforts of all parties involved in a supply chain to identify, assess, mitigate and monitor risks with the aim to reduce vulnerability and increase robustness and resilience of the supply chain, ensuring profitability and continuity.

The definition highlights both negative (unforeseen) and positive (predictive) connotations. A similar example exemplifies both types of connotations in Dumitrascu et al. (2020):

In supply chains, identifying, evaluating and monitoring risks represent key aspects that can reduce the vulnerability and increase the resilience of the supply chain, ensuring efficiency and sustainability. Risks refer to any threat of supply chains operations’ interruption and can be classified as operational, demand, supply or disruption risks.

Resilience as a form to mitigate disruptions, however, involves costs. Eliminating every potential threat would incur too high investments. Hence, resilience and flexibility can only be a measure to create a cost-effective flexibility configuration (Kersten et al. 2015). This match between eliminating uncertainty by increasing flexibility and/or redundancy is not a trivial task and involves cautious economic balancing.

Mitigation strategies and their success largely depend on how fast the information is delivered to decision-makers. In its first phase, preparation is the essential phase in which contingency plans may be drawn up. The key to successful decisions is that the sooner data is available, the longer the preparation phase is extended, or, more importantly, the sooner planned or normal production may resume. Decisions will always be more vigilant if more time is available. The more time is available between the actual event and its dissemination to the stakeholders, the better the alternative decision will generally be. The decay time is the phase with the greatest uncertainties since the length and descent of the curve are unknown. However, resilient supply chains—and whatever strategy may be behind it, e.g. diversification of suppliers, shifting inventory, stockpiling and reserves, guaranteed long-term contracts, swift switch of sources, re-routing shipments—minimise the drop and shorten the recovery time. The recovery time is the period to fully normal operations again. The overall time from the decay and recovery period is known as the total time to recover (TTR). Resilient supply chains lower the extent of the triangle on both axis (time and severity). There are various commercial systems on the market today that aim to support the minimisation of the resilience triangle by providing accurate and fast information feeds about disruptions in the supply chains and world markets. These applications are further introduced in Section 2.

Many data—weather, news, social media or company intelligence—today are distributed in real time or near real time. Generally, there is a wide range of data sources available for analysis. The next section will highlight pivotal data sources in big data analytics (BDA), starting off with a definition and the characteristics of big data.

### Definition and characteristics of big data

The type, size and speed of today’s available information vary greatly. Thus, big data is often referred to as the 5 Vs: velocity, variety, volume, veracity and value (Darvazeh et al. 2020). Velocity refers to the speed data exchanged, e.g. batch, near real time, real time, frequent or infrequent. As a matter of fact, data can have very different velocity. On one hand, valuable information needs to be supplied in real time in order for it to be useful, e.g. stock prices, traffic and logistics or weather. On the other hand, information may only come in once a year like macroeconomic indicators, e.g. GDP growth or political stability. Variety may be structured, semi-structured or unstructured information. This distinction is one of the pivotal characteristics of big data. Written text, news feeds, tweets, blogs, podcasts, streams and videos, pictures and satellite images come in very different formats and need to be analysed in completely different ways. Volume describes the size of information and is measured in bytes (and their ${10}^{3}$ steps). Volume is a key characteristic since it exponentially increases over time due to the increase in sources and their frequency. Raster images can today reach peta- (${10}^{12}$), exa- (${10}^{15}$) or zettabyte (${10}^{18}$). Value refers to the economic value inherent in big data. Veracity incorporates the validity of data sources with regard to growing concerns about the truthfulness and accuracy of information. Social media and also the internet suffer from a growing number of inaccurate or false information. The latter 2 Vs have been added as late as 2012 to the original 3 Vs. A definition of big data is put forward by Lamba and Singh (2017) who define it as:

data-sets that are so vast and complex that it is beyond the capabilities of traditional data management tools to capture, store, manage and process efficiently.

### Big data sources used in this study

#### Satellite data

Satellite data is a prime example of a continuously improved reconnaissance of the earth’s global surface. Remote sensing allows the observation of our planet with continuously enhanced spatial and radiometric resolution. Very high resolution satellites (VHR) today have a spatial resolution of 50 cm/pixel and better, e.g. WordView or GeoEye. Radiometric resolution refers to the electromagnetic wavelength recorded in different bands. The Sentinel-2 satellites record 13 bands with various radiation ranging from ultra blue (Coastal and Aerosol) to visible, short and near infrared. Satellites have become the most reliable source today to study climate change, observe sea temperature and water cycles like El Nino, ground level temperature and soil moisture, solar irradiance, depletion of the ozone layer, air quality, cloud structure, weather forecasting, wind, land use change, flora health, seismics, earth gravitational field, land slide monitoring, deformation studies, elevation and so forth. Even a night band exists on the Suomi NPP satellite to record the planet in darkness for scientific purposes, e.g. anthropogenic light pollution, population density or economic activity (NASA 2012).

For mineral raw material markets, satellite technology can be applied to forecast short- and mid-term weather phenomena that might have an impact on production and logistic networks. In this study, weather forecasting is an integral part of Everstream Analytics (2021) and used for tracking risks in mineral raw material supply. The data can in turn help to mitigate damage to mobile and stationary infrastructure, such as mines, transport of ores along roads and railways. Kayrros (2022), for example additionally uses a target monitoring based on satellite data, including (i) hear detection to track flaring activity and break outs of fire at industrial sites, (ii) fume detection to indicate operations, (iii) spill detection to avoid massive spills by a monitoring and alerting system, (iv) tracking of human activity to measure productivity of industrial sites and (v) 3-D modelling to measure stockpiles for mine products, among others.

#### Web scraping

The internet and information contained within are growing enormously. At current forecast levels, around 280,000 Petabyte (${10}^{18}$ Byte) a month are transferred globally through the internet (Cisco 2016). However, these numbers are growing rapidly and at current pace doubles in less than 4 years. Internet data is also prone to constant change through new and updated data. Any meaningful attempt to retrieve information on the internet can thus only be conducted with fully automated techniques and tools. The most commonly known method to search the internet is through web scraping.

Web scraping, also known as web crawling or web harvesting, is a special type of bots that searches the internet. There are various forms the internet may be searched. The most reliable source of data extraction is through application programming interfaces (APIs). These interfaces provide the extraction of data through standardised formats such as XML (extensible markup language) or JSON (JavaScript Object Notation). In the absence of APIs, websites have to be parsed. Parsing is the method that converts HTML source code into its written content by extracting text through automated structuring of the information contained within. HTML is a markup language (Hypertext Markup Language) containing content and additional information (e.g. attributes, styles, classes, id’s) about what the information contains in terms of styling and interpretation of the content. The additional information is referenced with tags in angle brackets that need to be removed. To only extract the content of the code means to strip the non-content information.

It is also possible to extract content through media monitoring tools. These tools scan text, audio and video sources from online media and/or social media. The challenge of web scraping is that global coverage needs to be polyglot, i.e. contains several languages. Mandarin (or Putonghua), for instance, needs to be included to account for China as the most important player in terms of both trade flows in the raw materials markets, exports and imports. To this end, it is evident why the term big data has been created to account for the huge variety not only with regard to data formats but also in terms of multiple languages.

Once the information is extracted, it can subsequently be further analysed with data mining and natural language processing (NLP) techniques, which is explained in more detail in the next section.

### Data mining and natural language processing

Natural language processing (NLP) is a branch of artificial intelligence (AI) with growing attention and importance. Text mining has become an important tool to cope with the influx of information found in the media. However, languages and their cultural implications and contextual nuances are not processed easily due to its many facets, e.g. humour, sarcasm, irony, conflicting meanings, dichotomies and oxymorons. Machine interpretation from text is very different to the human approach. But technology is catching up fast. The models can react to new, unknown data and classify them into categories or make predictions (Wenzel et al., 2019; for more details, see Towards Data Science (2018); Unite.ai, 2020). Today, there are countless applications to be found on the internet that have machine learning (ML) technology underneath, e.g. Google Search (word prediction), chatbots and translators (DeepL).

Artificial intelligence is in many cases an overrated term for how computers deal with digesting information. Rather, the term implies that the growing availability and reduced price in IT technology such as CPU, RAM and GPU facilitate the processing of more and bigger datasets. Nevertheless, its qualities should by no means underestimated. For example, text analysis techniques include word frequency, collocation, concordance, text classification, sentiment analysis, topic detection, language detection, clustering, keyword extraction and entity recognition (Hirata et al. 2020). Hirata et al. (2020) and Wenzel et al. (2019) provide a good overview of the various techniques applied to machines making sense to the written text. Baker et al. (2016) use newspaper analysis to assess policy-related economic uncertainty as one of the proxies in their economic policy uncertainty index (EPU index). Higher economic uncertainty has a negative correlation to investment and employment. A higher global uncertainty is also linked to a lower global and German industrial production (Ademmer et al. 2021). Online prices today are incorporated even by statistical offices to improve their forecasts for inflation and consumer confidence (Ademmer et al. 2021). News and press articles have a lot of potential in early indication of turning points in economic cycles and to predict these events more accurately and faster than traditional forecasting (Nowcasting).

There are currently numerous actors on the supply chain risk management market, e.g. Everstream Analytics GmbH (former DHL Resilience360), Riskmethods GmbH, Resilinc Corporation, Kayrros GmbH and Prewave GmbH. They all provide automated information systems to increase the visibility of incidents and information in the supply chain. However, their approach how this transparency is established varies according to the data sources utilised and their business strategies. The next section will introduce the analytical tool of the selected big data provider Everstream Analytics.

## Methodology

For this study, a wide range of incidents and events were tracked using the Everstream Analytics tool. Everstream Analytics is a supply chain risk management system developed by DHL. It implements a big data approach by means of the variety of data sources. Data for natural risks may, for example, stem from satellite data (meteorology data), earthquake data (earthquake observatories) or environmental data (country risk indicators; webcrawler) that may affect business operations. The meteorology data alone processes about 20.7 billion data points per day (Everstream Analytics 2021). The platform also uses its own and partners’ logistics network data to support the analyses. Ground, marine and aviation data are available from internal DHL data backed up by around half a million employees and collected from GNSS and barcode data. Other data sources are tapped through media monitoring tools, web scraping and natural language processing, including sources from journals, news or social media (see Section 2 “Data sources for big data analytics”). Together, they account for 1 million data sources in 25 languages that increase the transparency of the supply chain and their fragmented tiers.

### Country risk ranking

**Fig. 1:**
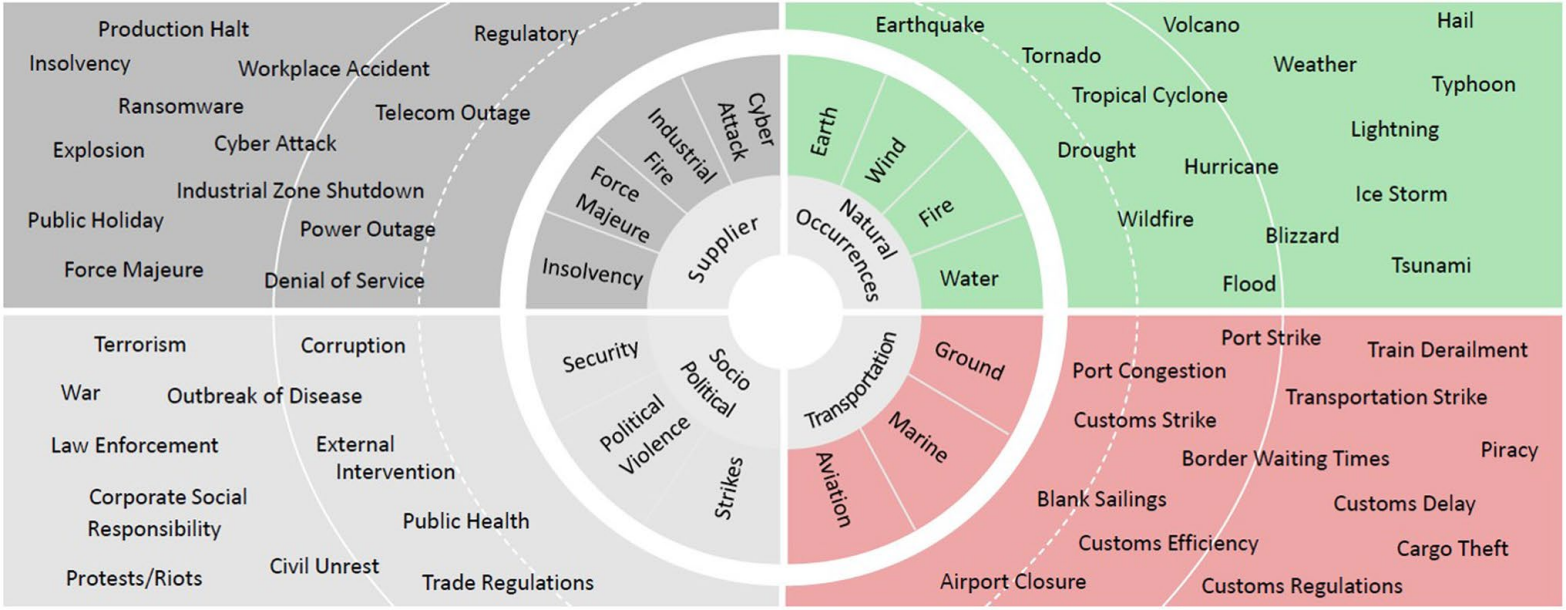
Risk categories and risk types monitored by Everstream Analytics for individual incidents (Everstream Analytics, 2021)

The Everstream platform further distinguishes between risk indicators of countries and two sources of incident tracking: supply watch and incidence monitoring. Altogether they cover a wide range of risk types (Fig. 1).

The country risk indicators are a collection of risk indicators from various public and internal sources. They cover a great range of relevant supplier, political, social, environmental, transportation and natural disaster risks or country-specific weather indicators, based on annual data or statistics. For comparison, the aggregated World Governance indicators by World Bank were used to measure the weighted average risk of countries involved in the production or trade of raw materials. Additionally, the global market concentration of mineral production was calculated by the Herfindahl–Hirschman index (see subsection “Adapted approach for this study and structure of analysis”).

### Supply watch

Supply watch is a web scraping approach which collects maximum media information for all locations of one network (here commodity type) in one day (without reporting the severity of incidents). The supply watch is a fully automated process. A vast amount of search results is of purely informational nature. Applied to the mineral raw material context, it means that the more media reports are published about a mining company or a mineral raw material, the more this firm might be in distress or the mineral raw material under public observation. Supply watch, in this respect, may act as an early-warning system to help identify increased public awareness and act upon emerging risks. The peaks are an indirect hint to the importance of events.

### Incident monitoring

Incidence monitoring is based on data mining and natural language processing but needs manual intervention to rate the severity of a single incident. The information is also scraped from a variety of sources, and the incidence creation and its classification of severity are supported by human intelligence provided by Everstream Analytics. In particular, the incidences need to be classified in terms of severity, i.e. minor, moderate or severe. This has huge implications on the warnings send out to customers. In return, customers can optimise the system to their risk profile. They have the chance to provide a feedback loop if the incidents are reasonably classified according to their original ranking. The ranking itself is provided by Everstream Analytics. However, the rankings are not generated by mineral analysts; so in this study, the results were verified and discussed by the authors and also to show how valuable the results are (see Section 5 “Discussion”).

Incidence monitoring distinguishes between two sources of incidents: exogenous and endogenous. The first group falls outside the control of normal business operations. These are the types of incidents that predominantly include particularly weather data and natural catastrophes, social and regulatory incidents. These incidents are outside the directly accessible and controllable supply chain. For these reasons, they are reactive in nature and need to be managed soon after occurrence if the supply chain is directly affected. In Fig. 1, they are visualised by the lower left “Socio-political” quadrant and upper right “Natural Occurrences” quadrant. The latter type of incidents—endogenous—directly affects the supply chain and can be more proactively managed. Since the control of incidents is much tighter linked to the supply chain, they are also more susceptible to predictive and prescriptive analytics. These incidents are displayed in Fig. 1 in the upper left quadrant “Supplier” and the lower right quadrant “Transportation”.

Incidents may need imminent solutions if disruptions become intolerable. Disruptions may not only have a severe negative economic impact, it may also be detrimental for the reputation of a company or the brand value, resulting in a shareholder value loss. For these reasons, rapid decisions need to be devised to diminish negative effects. In this regard, it is important to observe key performance indicators (KPI) such as stock prices of companies, financial performance or other relevant performance measures that may have an influence on an enterprise. This information is also vital to monitor the health and resilience of downstream suppliers in the chain. Supply watch and incident monitoring helps to identify companies that may cause a disruption or delay in the supply chain based on poor performance.

**Fig. 2:**
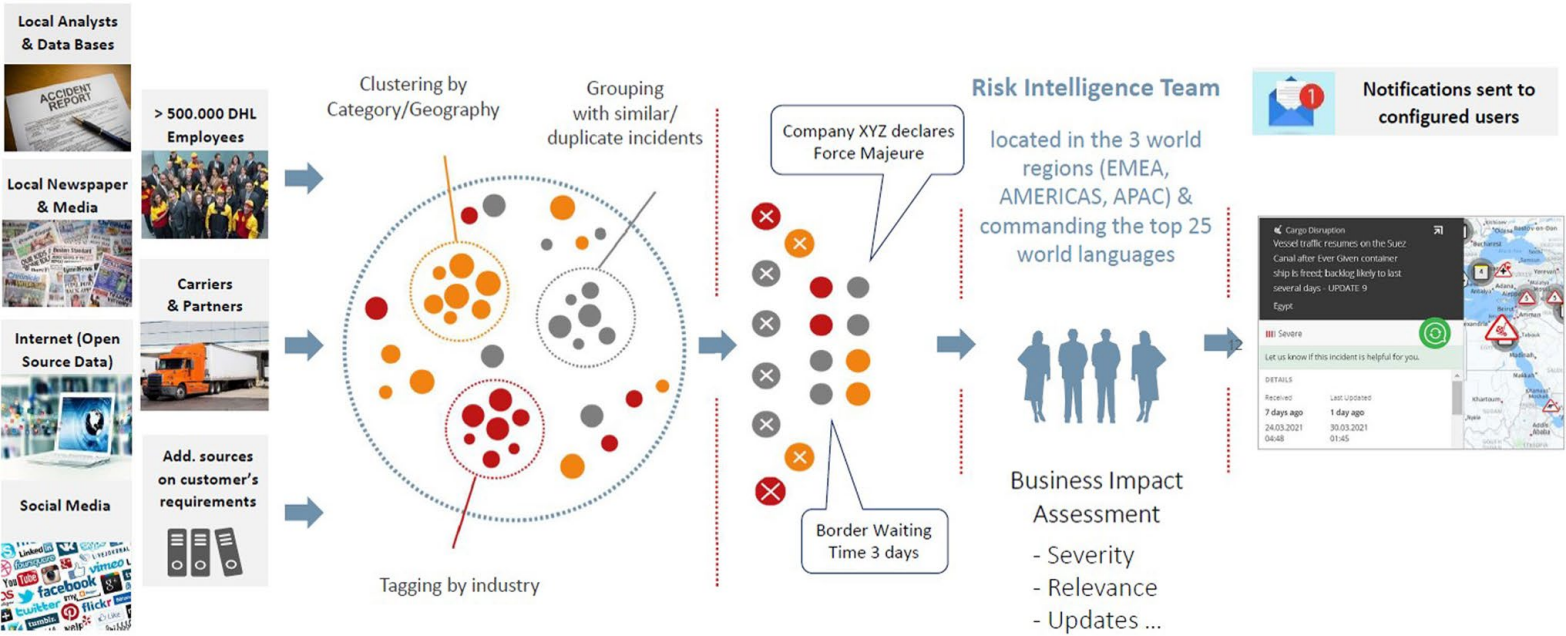
Everstream Analytics supply chain watch overview (Everstream Analytics, 2021)

Both, data from web scraping and incident monitoring, contain structured data as well as unstructured data that need to be transformed into groups and clusters. For the transformation, machine learning approaches are applied to prepare the data. Figure 2 exemplifies the transformation of big data into structured data that is used for further analyses and/or customer warnings. The transformation in addition with full or semi-automation in turn helps to increase the reaction time for incidents.

Data-driven decisions offer the opportunity to react to unforeseen events not only faster and with more time to find alternative strategies but also with more reliable information at hand. The time component enables decision-makers to react immediately if disruptions occur. The improved information also adds visibility to the various tiers of the supply chain. This transparency can help detect supplier risks earlier, improve performance in the supply chain by shifting resources where they are needed, e.g. in delayed estimated time of arrival (ETA), and help to identify corrective and preventive actions. Any changes or delays mean resources can be used elsewhere to increase the degree of capacity utilisation. Overall, information helps to improve performance in virtually all sectors of operational management from production to distribution and sales by allocating resources such as raw materials, labour, energy and capital equipment more efficiently.

### Adapted approach for this study and structure of analysis

A total of 12 mineral raw materials and the geolocations of important mines and headquarters were investigated, because comprehensive data was available for these mineral raw materials, namely, bauxite, cobalt, copper, graphite, iron ore, lithium, manganese, molybdenum, nickel, palladium, platinum and zinc. For these mineral raw materials, company data, production data and geolocations was provided by DERA (data source provided by S&P Global Market Intelligence, 2020b, used with kind permission). The database includes several thousand mine locations; however, only the major mines for each mineral raw material were considered. All major mines, each having a minimum share of > 1–2% of global production was tracked using Everstream Analytics (S&P Global Market Intelligence, 2020b, Data 2018, total: 280 mines & 90 headquarters). A share of < 1–2% of global production was defined as non-relevant to the market, because incidents at small mines may not strongly affect the global market. Following these requirements, 28.6 (zinc) to 97.5% (platinum) of global mine production were covered. The tracked mines are located in over 50 mining countries.

For the web scraping and incident monitoring exercise, a number of publicly available sources specialised for raw material markets were additionally included and tracked by Everstream Analytics, as demanded by the authors (mostly teaser tracking of daily news, including Asian Metal, Beijing Antaike Information, Benchmark Minerals, Bank of China Commodity Business Center, Fastmarkets, IHS Markit, Imformed Industrial Mineral Forums & Research, Roskill, Ruidow, Shanghai Metals Market, S&P Global Market Intelligence Metals and Mining, Wood Mackenzie).

In a first step, all 12 mineral raw materials were analysed using country risk indicators, the web scraping and the incident monitoring tools. The approach is to give an overview about the whole market and to show possible differences of risks between mineral raw material markets.

In a second step, out of these 12 mineral raw materials, five were selected as confident examples for a more detailed risk analysis. Only the most important incidents were described and summarised in this study, predominantly if the incident was relevant to the mining sector, had a national significance or possible impact on the mining sector, based on the authors’ expertise. The potential impact was quantified according to the global share of production at mines at risk.

Out of the five selected examples, platinum and palladium were grouped together in Section 4, but only for the incident monitoring results, not for the web scraping results. This makes sense for the following reasons:

- Web scraping searches for written content (text) and uses special types of bots that search the internet. They do not differentiate between various risk indicators. The media peaks and time series, which resulted from the analysis, were based on the searches for the written content “palladium” and “platinum”, separately.
- Incident monitoring searches for risk incidents, which occur at mine, regional or national level, are based on geographic coordinates or regions. Platinum and palladium are byproducts and thus are mined together. However, their share of production differs from region to region according to the geological setting. Risk events at platinum group mines thus always affect both raw materials, but the share of global production of these mines is different (see Table 4 for mines at risk; calculation is different for platinum and palladium).
- This study uses various levels of risks rankings:
- Market concentration of annual global mine production is based on the Herfindahl–Hirschman-Index (HHI, US Department of Justice and the Federal Trade Commission, 2010). In theory, higher concentrated market may react more sensitive to risk incidents than lower concentrated markets (for calculation see DERA, 2021a; HHI: 1–10,000; 10,000 highest risk; HHI: < 1500 = low risk; 1500–2500 = medium risk; > 2500 = high risk).
- Country risk is measured by the weighted average risk of countries involved in the production or trade of raw materials respectively and is based on the annual World Governance indicators (WGI) of the World Bank (2022, for calculation see DERA, 2021a). In theory, the higher the country risk is, the more risk incidents may occur (for calculation see DERA, 2021a; WGI: > 0.5 = low risk; 0.5-(-)0.5 = medium risk; < 0.5 = high risk).
- Country risk indicators are additionally provided by Everstream Analytics (various sources, mostly annual data), and the overall risk exposure of mine locations to individual country risk indicators was analysed (applied risk ranking: 1–100; 100 highest risk; risk ranking: < 30 = low; 30–50 = moderate; > 50 = high; > 70 = very high). The overall country risk ranking is the average of rankings of various country risk indicators by Everstream Analytics and sources therein.
- The Everstream Analytics tool covered web scraping of media posts for geolocations of important mines as well as of headquarters of mining companies in near real time (daily event or next day reporting) for the period January to June 2021 (limited time series available). The web scraping results were compared with the daily prices of five selected mineral raw materials to visually check if an incident had an obvious impact on the price.
- Mineral raw material prices were used based on various commercial sources. Price data are listed in the “DERA Price Monitor”, published on a monthly basis (DERA, 2021c). Daily prices were used for the selected raw materials including copper, zinc, iron ore, platinum and palladium.
- Incident monitoring of geolocations of important mines was provided by Everstream Analytics and covered the period June 2019 to June 2021. The severity types “extreme”, “severe” and “moderate” were applied. Minor incidents were neglected in this study because they are of minor relevance. The rating of the severity of incidents was provided by Everstream Analytics by manual intervention of Everstream Analytics staff.

Reference was given to all tracked incidents using public media sources (newspapers, magazines, social media, etc.) and first-hand company media releases. These are not necessarily primary sources, because all available big data tools are based on this principle of media screening, which is fast and effective.

## Results

### Overview and overall risks for the analysed mineral raw materials

The market concentration and the weighted country risk for the 12 mineral raw materials at country level range between low (HHI = 1182, copper; WGI = 0.25) and high (HHI = 5402, platinum; WGI = 0.06) values. Based on the Everstream Analytics country risk ranking, the average overall exposure of mine locations to country risk is moderate (43). Looking at individual risk indicators, operational and socio-political risks (corruption, law enforcement) are at the higher end (54 and 53) of that scale, and natural disaster (26; flash flood, tornado, earthquake) and political violence (29; civil unrest, war) risks are at the lower end of that scale. Risk to individuals (44; death and injury, detention) and sustainability risks (47; environment, workers’ rights) are at a moderate level. The best performing socio-political frameworks in which the studied mines operate were found in Canada, Australia and the USA. However, several individual mines operate in countries with a > 50 risk ranking, e.g. in Peru (natural disaster; sustainability), in Indonesia (operational, socio-political) or in Russia (very high for operational and socio-political risks).

### Web scraping results (January to June 2021)

**Fig. 3:**
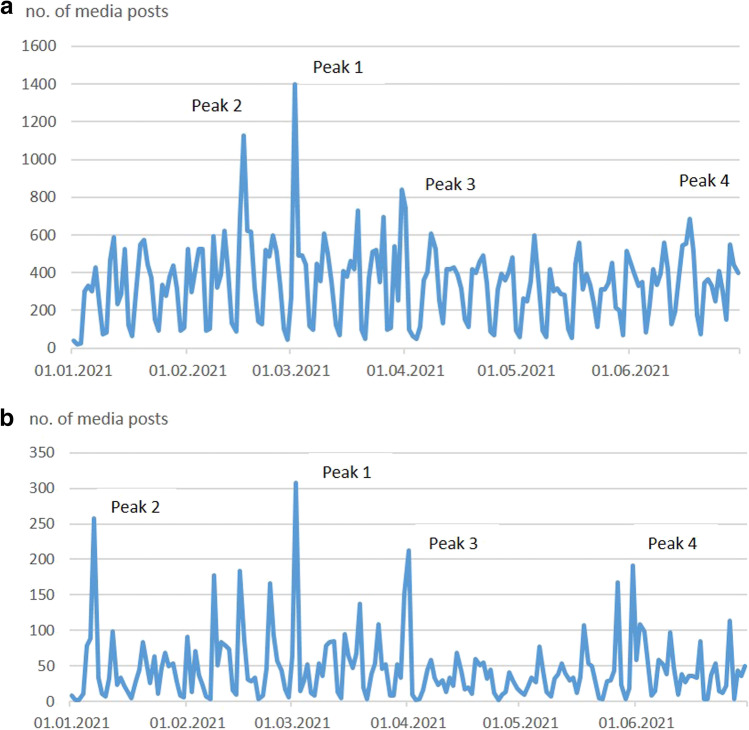
**A** Web crawler results for headquarters and mines for 12 mineral raw materials. Major peaks on a daily basis. Peak 1: Rio Tinto, Glencore, McArthur River (> 75%). Peak 2: Glencore, BHP, Teck, Norilsk Nickel, Rio Tinto (> 75%). Peak 3: Albermarle, Glencore, Mineral Resources, Gratomic, Wodgina Lithium, Arcelor Mittal, Silver Peak Lithium, Companhia Siderúrgica National, BHP Group, Rio Tinto (> 75%) (period: 01.01.2021–25.06.2021; location type: mines and headquarters; highlighted peaks: > 800 posts). **B** Web crawler results for mines for 12 mineral raw materials. Major peaks on a daily basis. Peak 1: McArthur River mine (> 73%). Peak 2: Las Bambas mine (> 90%). Peak 3: McArthur River mine, (Radomiro mine) (> 75%). Peak 4: McArthur River mine, Escondida mine (> 75%) (period: 01.01.2021–25.06.2021; location type: mines; highlighted peaks: > 200 posts)

Out of a total of 61,680 media posts for mines and company headquarters, the highest number of media posts falls in three risk categories: mergers and acquisitions, litigation and legal issues and governance issues (18.8%). About 50% of all posts were publications about the companies Glencore, Arcelor Mittal, Rio Tinto, BHP Group, Freeport McMoRan and Anglo American. The three major publication peaks (Peaks 1–3, > 800 posts) covered multiple news, events or incidents for several mining companies on a single day, covering a wide range of risk categories (Fig. 3A).

For mines only (8270 posts), the highest number of media posts falls in slightly different risk categories as compared to the analysis for mines and company headquarters, namely, mergers and acquisitions, litigation and legal issues and contamination/poisoning (13.9%). The highest amount of posts covered topics about McArthur River zinc mine (Australia), Las Bambas copper mine (Peru) and Los Pelambres copper mines (Chile) (36% of all mine-related posts). The three major publication peaks (Peaks 1–3, > 200 posts) point to major events at McArthur River zinc and Las Bambas copper mines (Fig. 3B).

### Incident monitoring (June 2019 to June 2021)

**Fig. 4:**
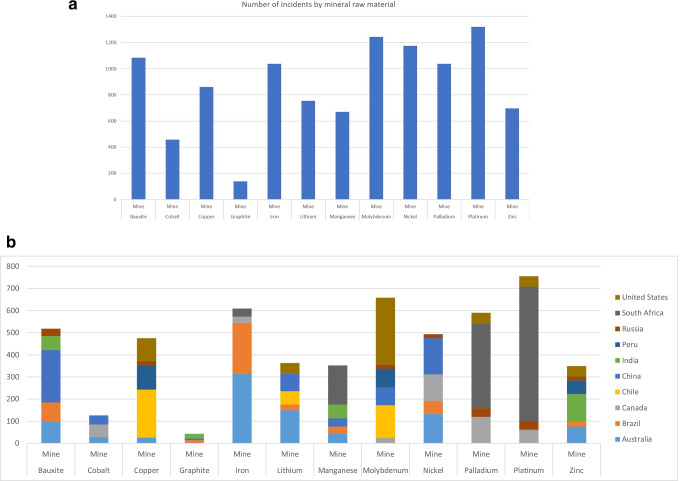
**A** Incident monitoring of mines for 12 mineral raw materials, total number of incidents by mineral raw material (Incident categories excluding “aviation”, “public holidays”, incident severity: moderate, severe, extreme; period: 20.06.2019–12.06.2021; location type: mines). Figure 4 **B** Incident monitoring of mines for 12 mineral raw materials, incident posts for top 10 countries (incident categories excluding “aviation”, “public holidays”, incident severity: moderate, severe, extreme; top 10 countries; period: 20.06.2019–12.06.2021; location type: mines)

Over the time period, a total of 10,479 incident media posts were reported for the 12 studied mineral raw materials. The highest number of incident media posts was for platinum, followed by molybdenum and nickel (Fig. 4B). The top 10 countries with the highest number of incident media posts were South Africa, followed by Australia, China, the USA, Brazil, Chile, Canada, India, Russia and Peru (Fig. 4B).

**Fig. 5:**
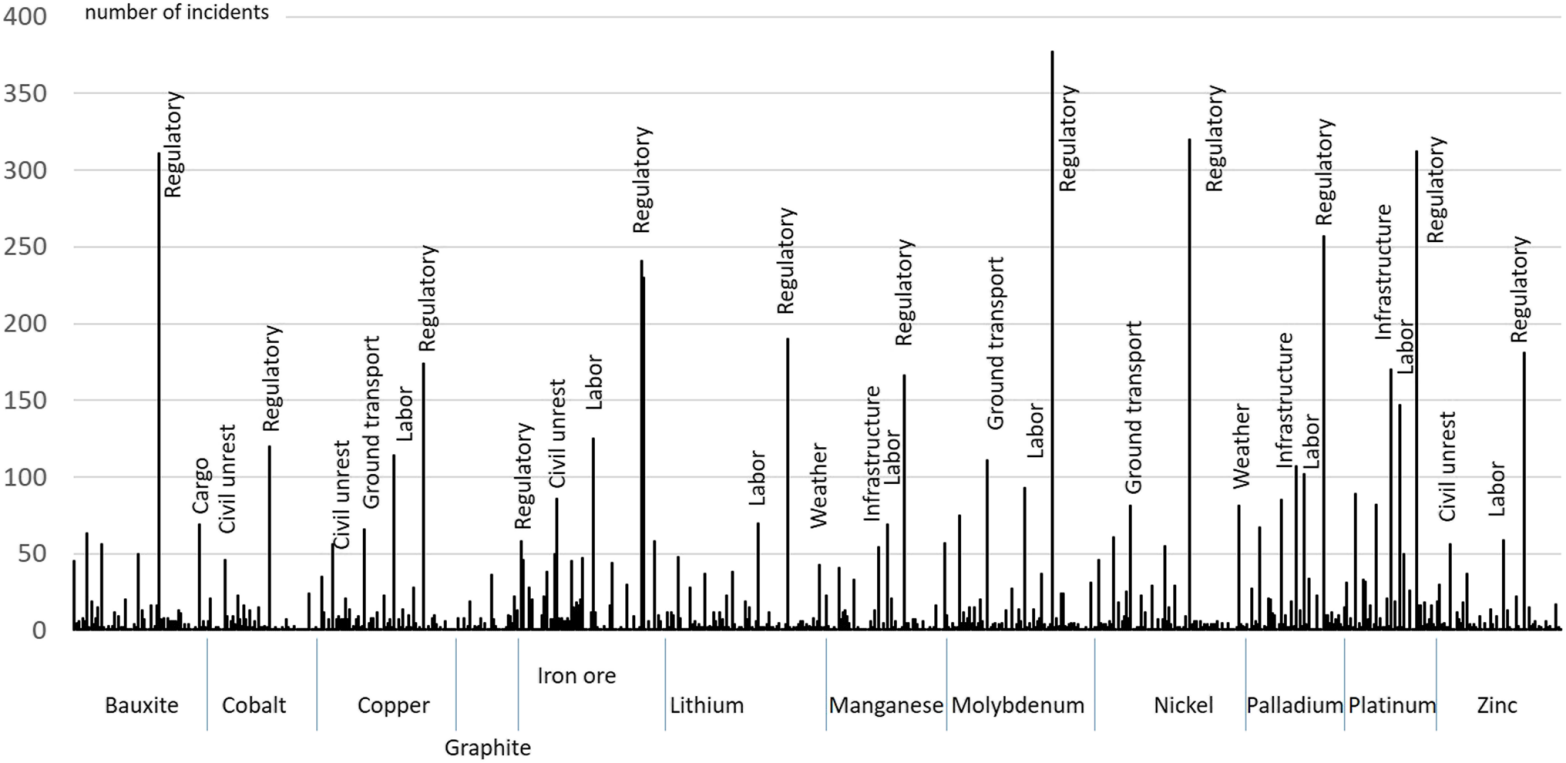
Incident monitoring of mines for 12 mineral raw materials, all incident categories (incident categories excluding “aviation”, “public holidays”, incident severity: moderate, severe, extreme; period: 20.06.2019–12.06.2021; location type: mines)

For all mineral raw materials, most of the incidents fell in the risk category of regulatory incidents, which mainly represent measures by governments or public authorities on country, provincial or communal level. Among those, COVID-19 measures were most prominent between 2020 and 2021. Other major incidents occurred in several raw material markets, namely labour, infrastructure, ground transportation, civil unrest or weather incidents (Fig. 5).

### Copper

#### Overview and country ranking

Copper is used as a conductive material in building and construction, domestic appliances, infrastructure, transport and industry (total 90%) (Dorner, 2020). The supply of copper is quite diverse and originates from over 20 mining countries. The five largest mining countries are Chile, Peru, China, DR Congo and the USA (total 59.9%, DERA, 2021c). For this study 24 mines covering 42% of the global production were analysed (Table 1) (mines > 1% of global production, S&P Global Market Intelligence, 2020b, Data 2018). The five largest copper mines worldwide are Escondida, Collahuasi (Chile), Grasberg (Indonesia), Cerro Verde (Peru) and El Teniente (Chile). El Teniente was not considered in the web scraping analysis because the name of the mine led to erroneous media posts (El Teniente means “the lieutenant” in the Spanish language).

**Table 1:** General indicators and web scraping results for five selected mineral raw materials; web scraping results for the period January to June 2021 (mines and headquarters; Everstream Analytics, 2021)

Mineral raw material	Share of global mine production covered by investigated mines [%]	HHI	Country risk (WGI)	Everstream Analytics overall exposure country risk	Total no. of media posts	First three strongest risk categories	No. of media posts	Highest no. of media posts, min. 50% of all posts	No. of peaks (peak benchmark)
Copper	42.0	1182	0.25	34.8	4090	Litigation/legal	772	Los Pelambres	3
(24 mines)						Taxation		Las Bambas	> / = 100
						Pollution/env		MMG HQ	
Zinc	28.6	1506	0.08	41.2	2925	Contamination/poisoning/ pollution	871	McArthur River	4
(16 mines)						Downgrade/underperformance		Nexa Resources	> / = 150
						Mergers & acquis		Zijin Mining	
Iron ore	57.0	2030	0.50	42	9466	Regulatory	8775	Arcelor Mittal	6
(23 mines)						Ground transportation		Fortescue	> / = 150)
						Civil unrest		Companhia Siderúgica	
Platinum	97.5	5402	0.06	40.7	13,916	Mergers & acquisitions	1275	Glencore	5
(21 mines)						Litigation/legal		Sibanye Gold	> / = 300
						Incident/accident		Vale	
Palladium	87.4	3178	− 0.04	40.7	135	Governance	82	Lac des Iles	5
(20 mines)						Labor practice		Unki	> / = 300
						Incident/disruption		East Boulder	

In general, the market concentration for mined copper at country level is low (HHI = 1182), and the weighted country risk is moderate (WGI = 0.25) (DERA, 2021a). Thus, the expected overall exposure of mines to country risk is relatively low (average: 34.8) (Table 1). The best performing socio-political frameworks in which copper mines operate are found at Mt. Isa (Australia), KGHM mines (Poland), mines in Chile (e.g. Chuquicamata, Collahuasi, Escondida, Radomiro Tomic) and Morenci mine in the USA (risk ranking < 30). None of the mines have an overall exposure to risk > 50. However, several mines in Peru have a > 50 risk ranking in certain risk categories, e.g. Toromocho, Antamina and Las Bambas, especially for earthquakes, civil unrest and flash floods (natural disaster; sustainability). High risk (> 50) are also ranked for Grasberg in Indonesia (operational, socio-political) or mines in Zambia (operational, socio-political, very high for sustainability) and in Russia (very high for operational and socio-political risk).

#### Web scraping results (January to June 2021)

**Fig. 6:**
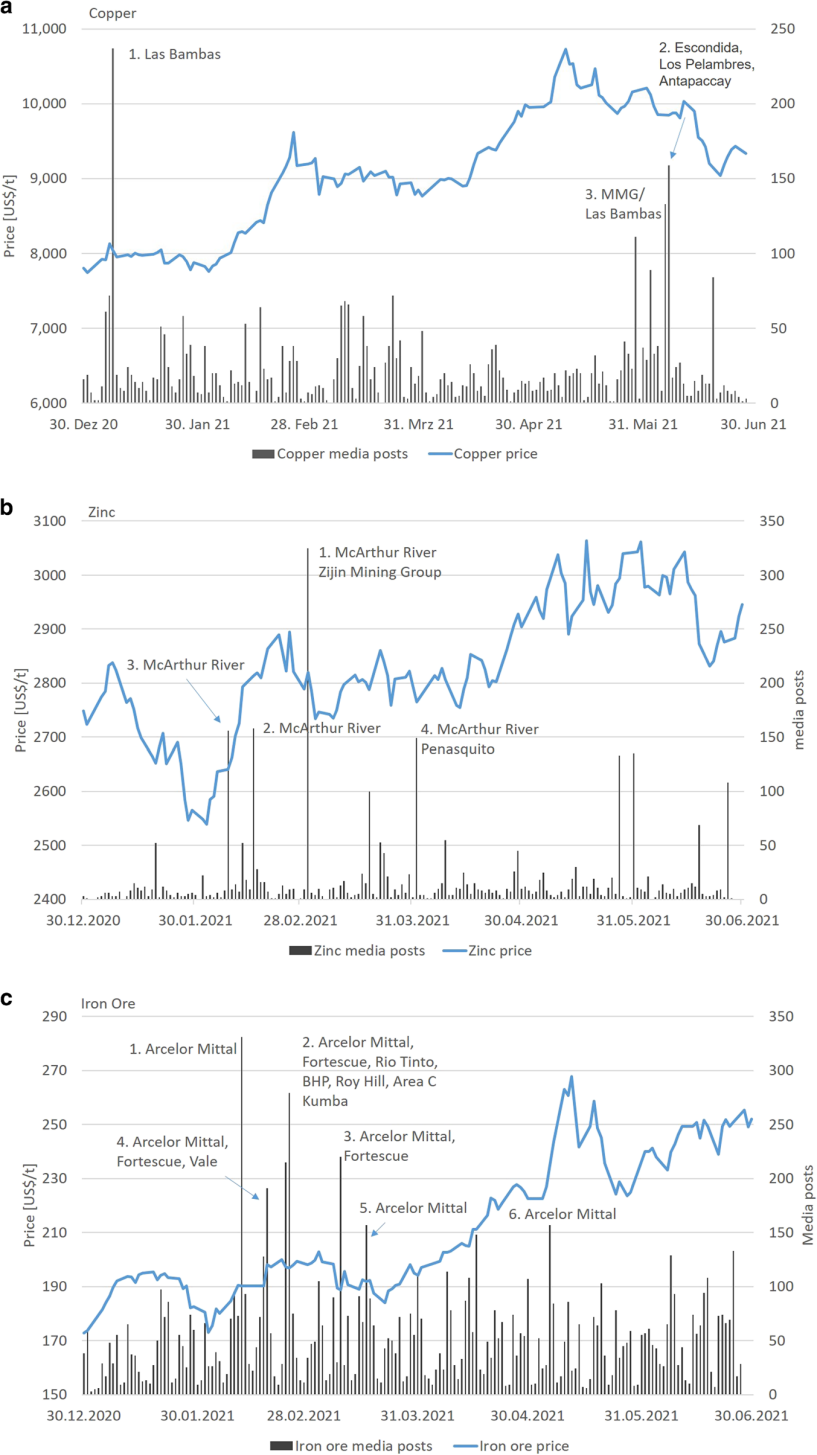

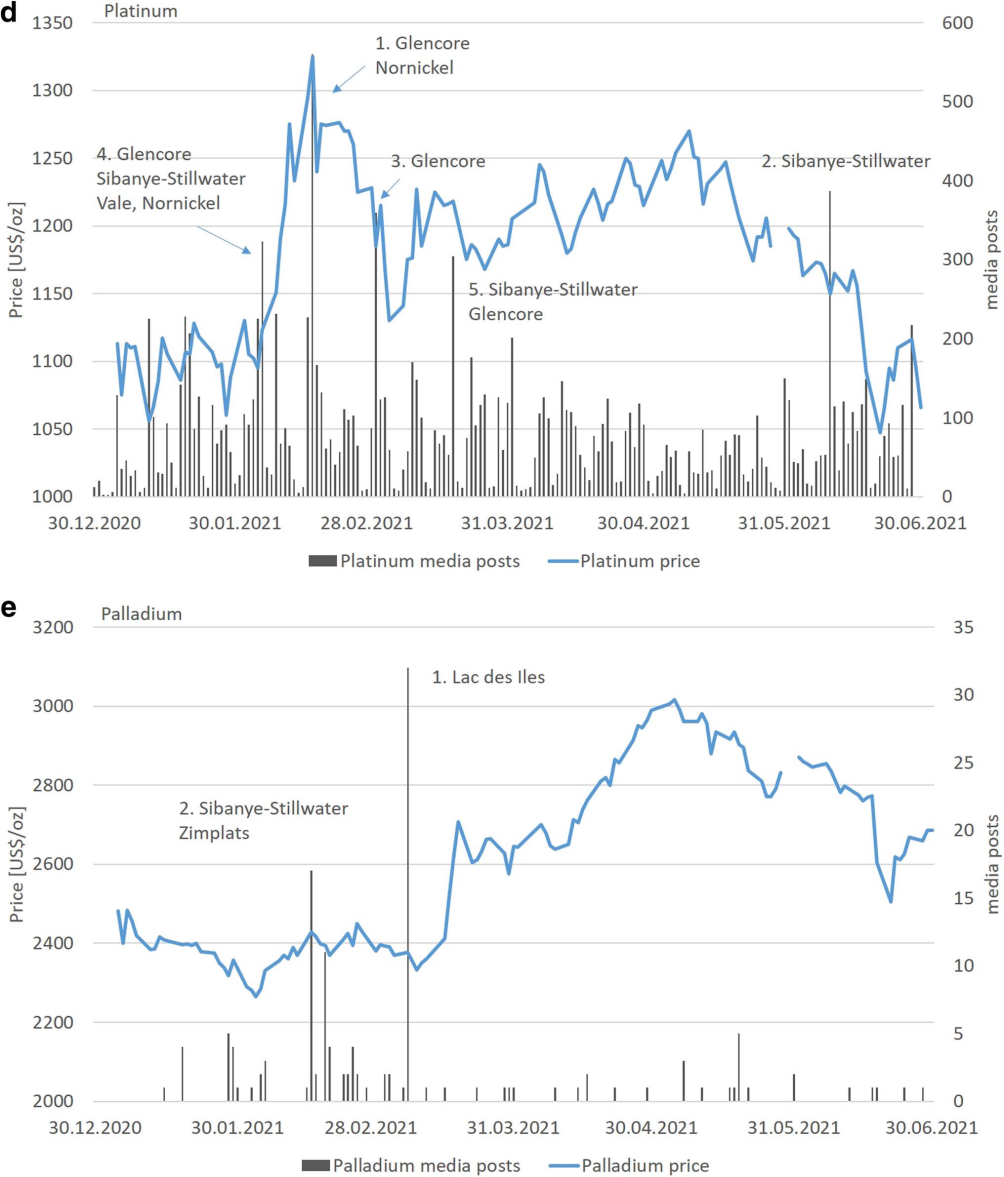
**a**–**e** Web scraping results for mine locations and daily price showing major media peaks for copper (**a**), zinc (**b**), iron ore (**c**), platinum (**d**) and palladium (**e**) (period: 01.01.2021–25.06.2021; location type: mines)

The highest number of media posts falls in three risk categories: litigation/legal issues, taxation and pollution/environmental issues (18.9%). Over 50% of all posts reported during the period relate to two mines (Los Pelambres, Chile; Las Bambas, Peru) and one headquarter (MMG, operating Las Bambas) (Table 1). The potentially affected share of global production by these two mines is 4.9%. Three peaks are prominent (Fig. 6a). Peak 1 may have caused an approximate 250 US$/tonne increase of the copper price from January 5 to 8, 2021, although only 1.8% share of global production was affected. None of the other peaks had a direct visual impact on the copper price. Events of Peak 3 could have affected 15.5% of global copper production, although the severity of the incidents and the possibility for supply disruption seemed to be rather low.

Peak 1, 05.01.2021, Peru: Blockades by local people for 3 weeks prevented the export of 189,000 tonnes of copper concentrates from Las Bambas mine (Reuters 2021a). The mine accounts for 1.9% of the global copper supply (S&P Global Market Intelligence, 2020b). Declining warehouse inventories at the London Metal Exchange and the Shanghai Futures Exchange combined with supply concerns from Peru led to an increase of the copper price according to media. Additionally labour, litigation and legal issues with regard to COVID-19 and other measures in Peru may have also impacted on the mining sector.

Peak 2, 07.06.2021, Chile, Peru: A number of events were reported from the copper market on that date. The union at BHP Billiton’s Escondida copper mine in Chile had submitted a preliminary proposal for a new collective wage agreement, marking the official start of wage negotiations at the world’s largest copper mine (threat of strike) (Reuters 2021f). At Los Pelambres (Chile), new electric vehicles were deployed at the mine (due to climate change concerns), and at Antapaccay mine in Peru, environmental mitigation plans were presented to the public (environmental issues) (DHL Resilience360).

Peak 3, 02.06.2021, Peru, Chile, USA: Presidential candidate of Peru, Pedro Castillo, had proposed to raise royalties on mineral sales and had announced tax renegotiation plans (inequality; taxation) which may affect copper mines in Peru such as Las Bambas and others (Reuters 2021e). In Chile, the mining group BHP had announced plans to spend up to $93 million in environmental repairs and reparations following a Chilean lawsuit related to BHP’s Escondida copper mine due to environmental damage in connection with overdrawing water from the Punta Negra salt flat (environmental damage) (Reuters 2021e). In the USA, a landslide inside Utah’s Bingham Canyon Copper mine disrupted operations (Table 2). However, mining operations resumed soon afterwards. No people were harmed, and no environmental impacts or damages to vehicles or structures occurred (Reuters 2021d).

**Table 2:** Results for web scraping of mines with > / = 1% share of global production plus web scraping of headquarters, January 2021 to June 2021

Commodity	Total no. of posts	First three stongest risk categories	No. of posts for strongest risk categories	Highest no. of posts for locations, min. 50% total of all posts	Potentially affected share of global production	Global production or price potentially affected (share of global production in %)											
						Peaks with > / = 100 posts in one day											
					[%]	Peak 1	[%]	Peak 2	[%]	Peak 3	[%]	Peak 4	[%]	Peak 5	[%]	Peak 6	[%]
Copper	4090	Litigation/legal		Los Pelambres mine	1.8	Las Bambas mine	1.8	Escondida mine	6.1	Peru mines	8.4						
		Taxation	722	Las Bambas mine	1.8			Los Pelambres mine	1.8	Escondida mine	6.1						
		Pollution/env		MMG HQ (total 1.3%; excl. Las Bambas)	0.5					Bingham Canyon	1.0						
Total					4.1		1.8		7.9		15.5						
						Peaks with > / = 150 posts in one day											
Iron ore	9466	Mergers & aquisitions		ArcelorMittal	2.1	ArcelorMittal*	0	Fortescue Group Metals	7.3	ArcelorMittal*	0	ArcelorMittal**	0	ArcelorMittal*	0	ArcelorMittal*	2.07
		Litigation/legal	1854	Fortescue	7.3			Area C mine	2.6	Kumba mine*	0	Fortescue (Western Australia)	7.2				
		Climate change/ carbon dioxide emissions		Companiha Siderúgica	1.1			Arcelor Mittal*	0			Vale (Brazil)	15.2				
							0		9.9		0		22.4		0		2.07
Platinum																	

^*^Incident/even does not potentially affect production

^**^Death of workers at steel plant in South Africa does not affect iron ore production but causes an image damage

#### Incident monitoring (June 2019 to June 2021)

Among all reported incidents for copper, only one extreme incident occurred (natural disaster/earthquake). The first three strongest incident risk categories were regulatory/advisory, civil unrest/security issues and labour issues. Most incidents for the tracked mines were reported for Chile, Peru and the USA (26.6% share of global production) (Table 3). Many incidents included a large number of reports related to the COVID-19 pandemic in 2020. In Chile and the USA, border closures/delays occurred between March and April 2020, with additional curfews and other restrictions imposed. Several other COVID-19-related incidents included production halts. For example, all larger mines in Peru were affected by COVID-19 at the beginning of the COVID-19 pandemic (8.5% share of global production; Table 3). Over the studied period, three larger mines with a maximum share of global production of 0.7% to 6.1% (highest Escondida) were potentially affected directly by other incidents at mine level, mostly for a very short period.

**Table 3:** Results for incident monitoring of mines with > / = 1% share of global production, June 2019 to June 2021 (incident categories excluding “aviation”, “public holidays”; incident severity: moderate, severe, extreme)

Commodity	Incident severety	No. of incidents	First three strongest risk categories	No. of incidents	Potentially affected first three countries with most incidents (local and nationwide incidents)	Share of global mine production [%])	Potentially affected mines (local incidents)	Major documented risk at mine level	Share of global production [%]
Copper	Moderate	474	Regulatory	147	Chile	18.1	Chuquicamata	Production halt at smelter and refinery (COVID-19)	1.6
(24 mines)	Severe + extreme	83	civil Unrest/sec	140	Peru	8.5	Escondida	Mine workers ‘ strike/threat of strike/environmental damage	6.1
			labor	101	USA	3.1	Grasberg	Production halt/reduced production (COVID-19)	2.8
							Bingham Canyon	Natural disaster/landslide	1.0
							KGHM (Rudna)	Natural disaster/earthquake	0.8
							Cerro Verde	Production halt/care and maintainance (COVID-19)	2.4
							Larger mines in Peru	Production halt (COVID-19), new taxation	8.5
							Las Bambas	Mine workers strike/road blockage and COVID-19 measures	1.9
							BHP (Spence)	Mine workers strike	0.7
Total		557		388		26.6			
Zinc	Moderate	347	Regulatory	150	India	29.7	San Cristobal	Production halt/suspension (COVID-19)	1.6
(16 mines)	Severe	63	ground transport/cargo	92	Australia	18.5	Tara	Production halt (COVID-19)	1.0
			Labor	40	Peru	14.9	larger mines in Peru	Production halt (COVID-19)	5.1
							Penasquito	Ground transport/demonstrations in the region	1.1
							McArthur River	Legal issue/cultural and environmental damage	1.9
							Yauli	Ground transport/natural disaster/landslide	1.1
							Kyzyl-Tash Turk	Ground transport/heavy flooding in the region	1.1
Total		410		282		63.1			
Iron ore	Moderate	541	Regulatory	167	Australia	33.0	Shipments via port Geraldton (Australia)	Weather/storm	< 0.1
(23 mines)	Severe	54	Labor	89	South Africa	1.2	Larger mines in the Iron Quadrangle (Brazil)	Production halt, civil unrest/demonstrations (COVID-19)	5.9
			ground transportation	82	Brazil	16.4	Shipments via Port Itaguai (Brazil)	Cargo disruption/port closure	1.9
							Marianna mine (part of Iron Quadrangle)	Production halt/environmental risk at Xingu dam	(1.1)
							Area C	Legal/conflict of interest/cultural heritage	2.3
							Ferrominera Orinoco	Disruptions by regulatory advisory (COVID-19)	1.1
							Saldanha Bay port (South Africa)	Port disruption (COVID-19)	1.2
Total		595				50.6			
Platinum	Moderate	165	Regulatory	343	South Africa	71.7	Anglo American Platinum mines (South Africa)	Production halt/force majeure (COVID-19)	24.3
(21 mines)	Severe	635	Labor	100	USA	9.6	Sibanye-Stillwater (South Africa)	Production halt/force majeure (COVID-19)	32.9
			Ground transport	87	Canada	3.5	Impala Platinum (South Africa)	Production halt/force majeure (COVID-19)	11.5
							Anglo American Platinum mines	Closure of Anglo Converter Plant Phase B unit	(4.9)
							Tharisa	Production halt (COVID-19)	1.4
							Booysendal	Production halt/force majeure (COVID-19)	1.9
							Rustenburg area	Civil unrest/riots in Rustenburg area	(> 40%)
							Zimplats/Ngezi	Accident/legal investigation	4.4
							Unki	Taxation/backlash	1.4
							Stillwater	Accident/legal investigation	9.6
							Nornickel (Polar Division)	Environmental risk/diesel fuel spill/legal investigation	4.2
Total		800		530		84.8			
Palladium	Moderate	516	Regulatory	261	South Africa	29.2	Anglo American Platinum* (South Africa)	Production halt/force majeure (COVID-19)	17.7
(20 mines)	Severe	119	Labor	75	Canada	11.4	Sibanye-Stillwater* (South Africa)	Production halt/force majeure (COVID-19)	6.8
			Ground transport	71	USA	2.5	Impala Platinum* (South Africa)	Production halt/force majeure (COVID-19)	1.2
							Anglo American Platinum mines***	Closure of Anglo Converter Plant Phase B unit	(3.5)
							Rustenburg area	Civil unrest/riots in Rustenburg area	12.3
							Zimplats/Ngezi	Accident/legal investigation	3.2
							Unki	Taxation/backlash	1.1
							Nornickel (Polar Division)	Environmental risk/diesel fuel spill	14.7
		635		407		43.1			

#### Extreme incident

In Poland, a 7.0 earthquake was registered near the KGHM Rudna mine on November 19, 2020. The earthquake resulted in the evacuation of 20 miners from the mine. However, no injuries or damages or major production halts occurred at the mine (DHL Resilience360).

#### Selected severe and moderate incidents

#### Peru

##### *Selected COVID-19-related incidents*

*Production halt, port and cargo disruption, regulatory advisory (severe and moderate)*: The Peruvian government imposed a mandatory quarantine of all residents from March 15 to March 30, 2020, as a preventive measure against the spread of the COVID-19 coronavirus. Production operations and cargo movement were expected to be severely affected (severe, regulatory advisory, CNN 2020). Some Peruvian ports were experiencing shortages of stevedores after the country declared an emergency status in March due to COVID-19. For example, Port of Matarani halted operations between March 19 and the end of March. ILO (Enapu) stopped operations on March 18, and the media reported that it may remain closed until the end of that month (severe, port disruption, 20.03.2020, DHL Resilience360 2020). Paita, Salaverry, Chimbote, Callao and Pisco were operating normally according to media reports. On April 3, the Peruvian government announced an amendment in gender-based movement restrictions (different days for men and women) with effect of April 13, due to the ongoing COVID-19 pandemic in those days. Only one household member was allowed to leave home for urgent issues and business between Monday and Saturday. No one was permitted to leave home on Sundays (until April 26, severe, regulatory advisory) (GardaWorld 2020). After further lockdowns in 2020, new COVID-19 lockdowns in 10 states of Peru were implemented from the end of January 2021 until February 14, 2021. The new lockdowns resulted in homeoffice work, the closure of all non-essential shops and the suspension of interregional land and air travel (moderate, regulatory advisory, Reuters 2021b).

*Production halt at mines (all rated moderate)*: Media reported on May 7, 2020 that all mines in Peru were operating at between 35 and 40% of capacity due to the COVID-19 pandemic (moderate; Reuters 2020n). Freeport-McMoRan announced on March 17 that it will put its Cerro Verde mine under maintenance for 15 days (production halt, S&P Global Market Intelligence 2020a). Cerro Verde copper mine still had limited capacity in early June 2020 projected until the end of June, 2020 (DHL Resilience360 2020). Glencore had announced that it will halt operations at Antamina mine for a period of 2 weeks also due to the COVID-19 outbreak (production halt, Reuters 2020b). Antamina mine was back at 80% capacity but with a reduced workforce in June 2020 (Mining.com 2020a). MMG announced on March 19 that it will cut operations at Las Bambas mine for 15 days (Fastmarkets, 19.03.2020). On June 4, MMG reported for Las Bambas mine that they would operate with “some fluctuations” because part of its workforce was not able to attend work due to mobility restrictions (DHL Resilience360 2020). In general, on June 10, the Peruvian government had authorised 91% of major mining operations to restart production after meeting COVID-19 prevention and control standards (11.06.2020).

##### *Other incidents*

*Labour, general strike, mine workers strike, ground transportation, protest/riot, roadway closure/disruption (moderate)*: Peru experienced increased local and nationwide labour strike activities, which affected some services and caused localised disruptions to production and transport. On March 19, 2020, the General Confederation of Workers of Peru (CGTP) had planned a strike over various issues, including the cost of living, the minimum wage and recent reforms in collective bargaining laws (DHL Resilience360 2020). Following this announcement, a dispute between government, mine workers unions and mining companies started over the nationwide COVID-19 measures. The nationwide measures did not include the mining sector, because it was declared an essential industry. Although mining companies were committed to “continue with a skeleton staff and [following] all the necessary health and safety precautions”, the National Miners’ and Metalworkers’ Union (FNTMMSP) stated that large mining companies would not follow those health and safety measures (IndustriAll 2020). Several labour protests followed by the end of November/beginning of December 2020. A nationwide strike by CGTP was planned for November 5, 2020, for the same reasons as in March (DHL Resilience360 2020). On December 1st, mine workers went on strike at Glencore mines over labour abuse (IndustriAll Union 2020). Miners also started a nationwide indefinite strike from December 4, 2020, joining farm workers strikes (Reuters 2020g). The strike was supported by the National Federation of Mining, Metallurgical, and Steel Workers, and the purpose of the strike was to protest against the “collective dismissal and work suspension procedures” (teleSur 2020). On November 30, 2020, protests in Challhuahuacho district disrupted transport of copper concentrate by road blockage from MMG’s Las Bambas mine. The strike was initiated by the Farmers’ Federation, the Defense Front and the Youth Federation (Mining.com 2020b) and lasted 24 days, after further negotiations “about financial contributions to the district for the road usage” broke down (Reuters 2020). The protests flamed up again in January 2021 (see Peak 1, web scraping results).

#### Chile

##### *Selected COVID-19-related incidents*

*Production halt, ground transportation, border closure/delay (severe and moderate)*: On March 22, Chile announced a nationwide night-time curfew due to COVID-19 (Reuters 2020a). On April 2, 2020, the Chilean government announced that it would close its borders for 1 month starting April 5 following a spike of COVID-19 cases. All international commercial flights were suspended (moderate, DHL Resilience360 2020). On June 26, 2020, Codelco stated that it would halt operations at its largest smelter and refinery in its Chuquicamata division as a preventative effort for safety and health (moderate, Reuters 2020d).

##### *Other incidents*

Between October to November 2019 and August to November 2020, a number of severe protests, riots and strikes over various issues affected Chile. The protests and violence flamed up October 18, 2019, in Santiago de Chile fuelled by rising cost-of-living pressures (The Guardian 2019). On August 2020, a severe cargo transportation strike was organised by truck drivers who announced a nationwide cargo transportation strike for August 27, if the Chilean government would not resolve and stop a growing wave of violent attacks in Southern Chile due to a lasting conflict between the State and the indigenous Mapuche group (Reuters 2020f). The strike came into effect on August 27 and ended a week later. In 2021, some strikes also affected mine operations. For example, mine workers at BHP Group’s Escondida and Spencer copper mines went on strike because the parties failed to reach an agreement to compensate 205 workers at the Integrated Operations Center for pits, cathode and concentrator plants (moderate, Reuters 2021c).

#### USA

##### *Selected COVID-19-related incidents*

*Regulatory advisory, ground transportation/border closure, cargo, postal disruption, production halt (severe)*: In March, 2020, severe COVID-19 measures were implemented in the USA. Most states declared a state of emergency and announced lockdowns to contain the COVID-19 virus outbreak, with non-essential businesses ordered to shut down. The hardest-hit states were declared disaster zones. A lockdown for 2 weeks was expected (regulatory advisory, U.S. Department of Defense 2020). Goodyear, for example, suspended all production in North and South America on March 18 until April 3, 2020. All facilities associated with tire, retreading and chemical operations including the mining sector were potentially affected (production halt, MarketWatch 2020). US authorities also announced border closures with Canada and Mexico in March 2020 for non-essential traffic. The land border closures were extended several times, e.g. until September 21, 2020, and until February 21, 2021, which might have affected also the delivery of spare parts to the mining sector (ground transportation/border closure, U.S. Department of Defense 2020).

##### *Other incidents*

*Regulatory/trade restrictions (severe)*: Between September 2018 and June 2019, US President Donald Trump announced that the USA would raise tariffs of Chinese goods from 10 to 25%, affecting a trade volume of USD 200 billion. China vice versa threatened to impose new tariffs on US goods worth USD 60 billion (BBC 2020a). On January 15, 2020, President Trump and Chinese Vice Premier Liu He signed an agreement as a first breakthrough of the nearly 2-year trade war between China and the USA. However, industry, including the mining sector, was advised to closely monitor the trade war negotiations. Trade disruptions continued with effect on March 16, 2020, when the Bureau of Industry and Security amended the Export Administration Regulations (EAR) that (additional) 24 entities were restricted from receiving US exports of goods (two entities in China, six in Iran, nine in Pakistan, two in Russia and five in the UAE). The reasons were involvement in nuclear-related activities, contributions to Pakistan’s missile program, transshipment of US-origin commodities to Iran and others (Federal Register 2020).

##### *Labor, cargo transportation strike, civil unrest, protest (moderate)*:

Truck drivers in the USA were discussing the possibility of a nationwide strike on November 29 to protest against COVID-19 lockdown measures and the outcome of the 2020 Presidential election (GobytruckNews 2020). On January 4, 2021, security reports were published about forthcoming nationwide protests in major cities in support of President Trump, who lost the 2020 election (G4S 2021). US House of Representatives and Senate had scheduled to certify the results of the Electoral College on January 6 in Washington, D.C. confirming Joe Biden as president-elect. Demonstrations on January 6 culminated in the storming of the U.S. Congress by Trump supporters caused chaos and turmoil in the capital during which 5 people were killed (CNN 2021b). The protest could have impacted ground transport and security issues nationwide, including the mining sector.

#### Other countries

After a rise in COVID-19 infections, Freeport-McMoRan stated on 18 May 2020 that it will operate its Grasberg mine in Indonesia with a “skeletal team” to avoid any further spread of the virus (production halt, Reuters 2020c). On August 24, more than 1000 mine workers blocked access to the Grasberg mine to protest against a COVID-19 bus services lockdown (moderate, labour strike, Reuters 2020e).

In Poland, a constitutional court ruling banning abortion sparked nationwide protests by the end of October 2020. The protesters blocked major streets, highways, rail lines and access to airports in several cities. They announced that they would not return to work until the court ruling would be overturned. Copper mines in Poland could also have been affected to a minor extent (moderate, general strike, The Guardian 2020).

### Zinc

#### Overview and country ranking

Zinc is mainly used for zinc and brass semi-manufactured products in the construction sector (41%), for galvanising in the automotive and construction sectors (39%), for chemicals and the pharma industry (11%) and for other applications (GDB, 2021). Zinc is mined in over 20 countries. The five largest mining countries are China, Peru, Australia, the USA and India (total 65.5%, DERA, 2021c). For this study, the 16 biggest zinc mines covering 28.5% of global production were analysed (S&P, 2020, Towards Data Scienceata 2018) (Table 1). The five largest zinc mines worldwide are Red Dog (Alaska, USA), Rampura Agucha (India), Antamina (Peru), Kazzinc (Kazakhstan) and Mt. Isa (Australia).

In general, the market concentration for zinc at country level (HHI = 1506) and the weighted country risk are moderate (WGI = 0.08) (DERA 2021a). Thus, the overall exposure of mines to country risk is moderate (average: 41.2) (Table 1). The best-performing socio-political frameworks in which zinc mines operate are at Tara (Ireland) as well as Mt. Isa and Dugald River (Australia) (< 30). Several mines have a risk ranking of 70 and higher in certain risk categories, for example, Kyzyl-Tash Turk mine in Russia for operational risk, social political risk, cargo theft, corruption, customs inefficiency, detention and discrimination, floods and law enforcement. Antamina and Yauli mines in Peru are sensitive to civil unrest, flash floods and earthquakes, other mines in Peru for earthquakes only. Bisha mine in Eritrea operates in an environment of elevated civil unrest, sustainability issues, corruption, customs inefficiency, detention and discrimination and problems with law enforcement and workers’ rights. Kazzinc in Kazakhstan has a high-risk exposure to corruption, environment and law enforcement. Other mines in Brazil, Bolivia and India have high risks in selected risk sub-categories as well.

#### Web scraping results (January to June 2021)

The highest number of media posts falls in three risk categories: contamination/poisoning, downgrade/underperformance and mergers and acquisitions (30.0%). In general, each major peak results from one or multiple incidents. For zinc mining, four peaks are prominent (ca. > / = 150 posts in 1 day, various languages, each peak covers various risk categories, Fig. 6b). The media posts mainly cover reports about Australia’s McArthur River lead–zinc mine operated by Glencore (Table 1). A direct impact on the zinc price was not found (Fig. 6b).

Peak 1, 02.03.2021: Expansion of zinc production at McArthur River Mine could have impacted indigenous sacred sites due to a growing waste rock pile that came 35 m close to the boundary of the site (Mining.com, 2021a, b). The community raised questions about the maintenance and protection of the sites on the mining lease and access to them. This happened against the background that iron ore miner Rio Tinto destroyed 46,000-year-old caves at Juukan Gorge in Western Australia subsequent to mine expansion in May 2020, which caused international criticism. The event caused serious questions about the maintenance and protection of sacred sites by mining companies in Australia.

Additionally, a larger number of media posts referred to the mining headquarter Zijin Mining Group, also a zinc miner, which announced the start of commercial production at its copper–gold Timok mine project in Q2 2021 in Serbia (Minenportal, 2021).

Peak 2, 15.02.2021: Local communities launched legal action against the Northern Territory government to challenge the decision of November 2020 to lower the McArthur River Mine’s environmental security bond by nearly $120 million (The Guardian, 2021). The communities protested against environmental incidents and irreversible cultural and ecological damage at sacred Indigenous sites from the mine operation.

Peak 3, 08.02.2021: The University of New South Wales (UNSW) and the Environmental Centre of the Northern Territory (ECNT) published a new report about the environmental impact of McArthur River mine. The report warned that up to 22 sacred indigenous sites and an important river system would face “irreversible cultural and ecological damage” from the mining operation. Northern Territory Ministry of Mining and Industry approved an amended mining management plan in 2020 which allowed Glencore to continue mining that would “minimise environmental impacts and ensure long-term beneficial land uses after closure” (miningweekly.com).

Peak 4, 01.04.2021: Glencore entered talks with the Northern Land Council and native title holders to negotiate an Indigenous Land Usage Agreement for McArthur River mine and the Bing Bong loading facility on the Gulf of Carpentaria. The talks included sacred sites and cultural heritage protection plans around the mine (7News.com, 2021). Some minor amount of posts covered an announcement by metal miner Newmont Corporation about their first quarter 2021 earnings including Penasquito gold-silver-lead–zinc mine in Mexico (Newmont Corporation, 2021).

#### Incident monitoring (June 2019 to June 2021)

Reported severe and moderate incidents for zinc include a large number of reports related to the COVID-19 pandemic in 2020. Most incidents were regulatory advisory measures, ground transportation and labour issues (Table 3). Several severe incidents include production halts due to COVID-19. Most incidents for the tracked mines were reported for India, Australia and Peru (63.1% share of global production). Although China is by far the biggest zinc producer at mining and refining stages globally, the biggest mines are located outside of China (S&P Global Market Intelligence, 2021). Thus, incidents for zinc in China were not covered in this study. For the studied period, all larger mines in Peru (5.1% share of global production) and some in other countries were all affected by COVID-19 at the beginning of the COVID-19 pandemic. Three larger mines with a maximum share of global production of 0.7 to 1.1% (highest Yauli) were potentially affected by other incidents at mine level, mostly for a very short period.

#### Selected severe and moderate incidents

#### USA

COVID-19-related incidents (see section “Copper”).

#### Australia

Selected COVID-19-related incidents and large-scale *cyber attack on June 19, 2020 (see iron ore).*

#### Bolivia

##### *Selected COVID-1-related incidents*

*Production halt (severe)*: On March 26, Sumitomo Corp announced that it would temporarily suspend operation at San Cristobal silver-zinc-lead mine to prevent the spread of the COVID-19 coronavirus (Sumitomo Corporation, 2020).

#### Peru

COVID-19-related nationwide incidents (see section “Copper”).

##### *Production halt (severe and moderate)*

Production halt (severe and moderate): As mentioned for copper, media reported on May 7, 2020, that all mines in Peru were operating at between 35 and 40% of capacity due to the COVID-19 pandemic (moderate; Reuters 2020n). On April 14, 2020, Glencore announced that it would halt operations at Antamina zinc-lead-copper mine in Peru for a period of 2 weeks due to the COVID-19 outbreak (severe; NS Energy 2020). On May 27, Compania Minera Antamina announced that it would increase its Antamina zinc-lead-copper mine to 80% capacity and a reduced workforce, after Peru’s government allowed mines to resume larger-scale operations (Metal Bulletin 2020). At least 41 large mining companies were hit by the COVID-19 measures in Peru (Reuters 2020o).

##### *Natural disaster, ground transportation, landslide, roadway closure/disruption (moderate)*

A landslide causing substantial disruptions on Highway 22, south of San Mateo in Peru, caused substantial disruptions as the route was blocked. The estimated time for reopening was unclear (DHL Resilience360, 2021). The road is the main connection from Yauli zinc mine to the west coast.

#### India

##### *Selected COVID-19-related incidents*

*Regulatory advisory (severe and moderate)*: India’s government announced on March 24, 2020, that the entire country would be locked down for 3 weeks in an effort to stop the spread of the coronavirus. This impacted domestic airport operations, shipments to and from port and airport terminals and inter-city and inter-state transport due to driver shortages (The Hindu 2020). On April 22, 2021, Indian authorities banned the supply of oxygen for industrial purposes due to severe shortages of oxygen for medical purposes amid COVID-19 surge (moderate; CNN 2021a).

*Regulatory, aviation, ground transportation, health (moderate)*: On April 30, 2021, the US government restricted travel into the country from India starting May 4 as India became the epicentre of the pandemic. Other countries authorised similar measures (CNBC 2021).

##### *Other incidents*

*Regulatory, trade restriction, customs regulation (moderate)*: On June 18, 2020, the Indian government announced regulations to block imports of cheap, sub-standard goods from China and other countries. About 25,000 products were affected (The Hindu^ 2020^). On July 13, media reported that India was considering to raise primary customs duties and non-tariff measures. A list of 1173 items including auto parts, compressors for AC and refrigerators, steel and aluminium products and electrical machinery was under consideration, which would also affect the mining industry (Grain Mart India 2020). These measures were in light with an app ban by China and ongoing military border clashes between China and India that have resulted in various casualties.

*Security, ground transportation, labour, public safety/security, roadway closure/disruption, train delays/disruption, miscellaneous strikes (moderate)*: A nationwide shutdown strike was planned by farmers from multiple organisations for September 25, 2020, due to several controversial agricultural ordinances. Associated demonstrations previously had caused major disruption (Garda World 2020a).

#### Ireland

##### *Selected COVID-19 related incidents*

*Production halt (severe)*: Boliden AB halted production on March 28, 2020, at Tara zinc mine for at least 14 days due to COVID-19 restrictions by the Irish government. Boliden also evaluated how the loss of production at Tara mine could be compensated by other sources of concentrate supply for its zinc smelters (New Boliden 2020).

##### *Other incidents*

*Weather, infrastructure, flooding, power outage, storm, weather advisory (moderate)*: On August 20, 2020, the Irish Meteorological Service warned of very severe and destructive winds and heavy rain until August 28 throughout Ireland as Storm Ellen would cause damages and flooding (The Journal 2020). Heavy rains were anticipated to persist throughout Ireland until August 28 following passage of Storm Francis, which may affect the Irish infrastructure.

#### Kazakhstan

##### *Selected COVID-19-related incidents*

*Regulatory advisory (severe)*: Kazakhstan authorities implemented state of emergency and quarantine measures on March 15, 2020, due to COVID-19 (Reuters 2020p). Quarantine and lockdown measures were imposed on a number of other cities and regions across the country. With effect of March 30, six cities were under quarantine. Residents were restricted from leaving their homes, except for food, medicine, banking and work (DHL Resilience360 2020).

*Cargo, ground transportation, cargo disruption, border closure/delay (moderate)*: Since October 2020, road and rail freight movement at the border with Kazakhstan was limited due to China’s COVID-19 restrictions. The restrictions greatly affected export and import. About 7500 trucks stranded for up to 42 days at the Dostyk-Alashankou border crossing according to media reports on January 6. Capacity reductions via truck and rail freight were expected for the first quarter 2020. Further border closures or restrictions on short-notice were possible due to COVID-19-related lockdowns in the neighbouring Xinjiang Uygur autonomous region (DHL Resilience360, 2021).

#### Mexico

##### *Selected COVID-19-related incidents*

*Regulatory, ground transportation, regulatory advisory, border closure/delay (severe)*: On March 31, 2020, Mexico declared a health emergency and issued stricter rules due to the COVID-19 pandemic until April 30. However, the country had not ordered a full lockdown due to concerns that Mexico’s economy would slump further, e.g. exempting cargo movement (Reuters 2020q). On May 20, 2021, US authorities announced that the shared border with Mexico would be closed to non-essential travel until June 21, 2021 (Reuters 2021n).

##### *Other incidents*

*Civil unrest, ground transportation, security, protest/riot, roadway closure/disruption (moderate)*: On May 30, 2020, anti-government demonstrations in several major cities occurred nationwide which demanded the president to step down. Protesters were blocking major roadways, and it was unclear how long the protests were planned for (The Statesman 2020). On June 5, additional protests were reported in Mexico City and Guadalajara following civil unrest after the death of Giovanni Lopez under police custody in the state of Jalisco. Additional protests were likely to take place in key urban centres in the following days. The Penasquito zinc mine lies in the state of Zacatecas, neighbouring the state of Jalisco (Mexico News Daily 2020). On September 28, 2020, nationwide Women’s Rights Demonstration led by multiple women’s rights organisations was announced as they demand the legalisation of abortion (Reuter, 2020r). For all protest, disruption in ground transportation was anticipated.

##### Production halt, fuel disruption, power outage, trade restrictions, infrastructure, regulatory (moderate)

On February 18, 2020, the Mexican industry was impacted nationwide by a natural gas shortage and power blackouts caused by a winter storm and cold temperatures in the northern part of Mexico and the US states Texas and New Mexico which declared force majeure (Fronteras 2021).

#### Russia

selected COVID-19-related incidents (see platinum group elements).

##### *Other incidents*

*Weather, ground transportation advisory, regulatory advisory, flooding (moderate)*: On July 20 2020 authorities of the Tuva Republic limited the movement of people within the republic for an indeterminate number of days due to heavy flooding. The flooding could have affected freight transportation (DHL Resilience360 2020). The Kyzyl-Tash Turk zinc-lead-copper mine lies in the Tuva Republic.

### Iron ore

#### Overview and country risk ranking

Iron ore is mainly used to produce steel and ferroalloys in buildings and infrastructure (52%), automotive, metal products, mechanical equipment (total 90%) and other applications (World Steel Association, 2021). The supply of iron ore is from over 20 mining countries. The three largest mining countries are Australia, Brazil and China (total 69.9%, DERA, 2021b). For this study, 23 mines covering 57% of the global production were analysed (mines > 2% of global production; S&P Global Market Intelligence, 2020b, Toward Data Science 2018) (Table 1). The five largest iron ore mines worldwide are Hammersley (Australia), Serra Norte (Brazil), Chichester, Newman and Yandi (Australia).

In general, the market concentration for iron ore at country level is moderate (HHI = 2030) and the weighted country risk is low to moderate (WGI = 0.50) (DERA, 2021a). Thus, the expected overall exposure to country risk is rather moderate (average: 42) (Table 1). The best performing socio-political frameworks in which iron ore mines operate are Mont-Wright (Canada), Jimblebar, Wheelarra Lease, Newman, West Angelas, Area C, Chichester Hub, Hope Downs and Roy Hill (Australia) (risk ranking < 30). Only one mine has an overall exposure to risk of 50, which is Ferrominera Orinoco in Venezuela. Several mines in Australia have a > 50 risk ranking in certain risk categories: Yandi, Robe River (Pannawonica) and Solomon Hub (natural disaster) and Hammersley (flash floods), as well as Sishen in South Africa (operational, socio-political, risk to individuals) and mines in Brazil (very high for operational, risk to individuals, flash floods for Casa de Pedra).

#### Web scraping results (January to June 2021)

The highest number of media posts fall in three risk categories: mergers and acquisition, litigation/legal issues and climate change/carbon dioxide emissions (19.6%). Over 90% of all posts reported during the period relate to three mining headquarters (ArcelorMittal, Fortescue, Compniha Siderúgica) (Table 1). For iron ore mining, six peaks are prominent (Fig. 6c). A slight price increase of 5% on February 17/18 (Peak 4) might be in connection to shipping risks according to weather impacts at ports in Australia. On May 6, 2021 (Peak 6), a strong price increase of 17% could have been supported by a positive market sentiment for iron ore and steel based on the global COVID-19 economic recovery, expressed by the strongest financial result achieved by ArcelorMittal for the past decade. Incidents reported for Peaks 2 and 4 could have affected companies, which control 10 to 20% of global iron ore production, or important ports for a short time.

Peak 1, 11.02.2021: 98% of all posts for Peak 1 cover reports about a change of management at ArcelorMittal, the world’s leading integrated steel and mining company. The company announced that Aditya Mittal, the company’s chief financial officer and son of Lakshmi Mittal (family majority owner of the steel making giant), would replace his father as CEO. ArcelorMittal promoted a generational change due to rising domination of the industry by China and growing investor pressure to produce steel more CO_2_-clean (governance, management, climate change; S&P Global Market Intelligence 2021).

Peak 2, 24.02.2021: Fortescue Metals Group cleared land on a heritage site for iron ore mining in Western Australia (WA), despite pressure on Australian iron ore miners to improve the management of important heritage sites. Back in May 2020, Rio Tinto destroyed an ancient Aboriginal rock shelter at Juukan Gorge for mine expansion, which caused a public outcry. Although Fortescue had the WA government permission to clear the land in the Weelamurra Creek, the company did not involve community elders “to perform salvage and cultural rites” (cultural risk; Reuters 2021h). Another incident at a heritage site occurred near BHP Groups’ Area C iron ore mine. BHP Group launched a joint investigation with an indigenous group to examine a rock fall at a culturally significant rock shelter of the Banjima people on January 29, as part of its monitoring at Mining Area C operations (Reuters 2021i). Area C is part of BHP’s $3.4 billion South Flank replacement project in the Pilbara state (accident, cultural risk, conflict of interest). Additionally, two major reports about ArcelorMittal were part of elevated media post: the sale of 3.76 million ArcelorMittal shares by the Mittal family (transparency; Delano 2021) and a forecast that ArcelorMittal would post sales of $15.57 billion for Q1, 2021 (Mergers & Acquisition; Litigation/Legal issues; DHL Resilience360, 2021).

Peak 3, 10.03.2021: A number of events caused this peak. Media posts included (1) an announcement by ArcelorMittal, which is pushing the industrial production of carbon neutral steel (climate change; Reuters 2021j), and (2) seasonal weather factors for iron ore shipments in Australia with minor effect, along with Saldanha port problems in South Africa. The shipments at Saldanha port fell 11.2% month on month in February (disruption/delay/setback); (3) message about the retirement of Mandla Gantsho, chairperson of Kumba Iron Ore mine in South Africa, which is a member of the Anglo American PLC group (management/leadership changes).

Peak 4, 18.02.2021: More than 50% of the media posts relate to an accident at a steel plant of ArcelorMittal in South Africa. The death of three workers at Arcelor Mittals Vanderbijlpark plant in Gauteng, South Africa, was confirmed on February 18 (SABC News 2021a). The workers were trapped under rubble following an explosion at the plant. The National Union of Metalworkers of South Africa (NUMSA) called the Department of Employment and Labour to carry out a detailed investigation into the cause of this incident (incident/accident; Misconduct/Negligence; Safety; Workplace accidents; Union activity). Another headline dominated that day: Fortescue Metals Group Chief Executive Elizabeth Gaines reported that “not only in China, but the ex-China activity is also picking up to pre-COVID levels”. She stated that the market would remain robust for some time, given supply concerns and stronger demand from China. Ongoing supply issues included COVID-19-related disruptions at Brazil’s Vale operations and the potential for further weather-related port stoppages at iron ore terminals in Western Australia. Fortescue, for example, exports iron ore from the world’s largest hub Port Hedland, Western Australia, where storms already halted operations twice (discontinue/suspend; Reuters 2021g). Iron ore exports in February 2021 were about 5 million tonnes less compared to iron ore exports in January 2021 (Pilbara Ports Authority 2021a). Port Hedland was closed for 16 h in February 2021 due to Tropical Low 12U (Pilbara Ports Authority 2021b).

Peak 5, 17.03.2021: Almost 90% of all media posts of that day covered an announcement of ArcelorMittal. The company launched three low carbon branded initiatives to provide customers with products to address their own carbon reduction targets: “(1) Groundbreaking ‘XCarb`™ green steel certificates offering customers Scope 3 emissions reductions (2) ‘XCarb™ recycled and renewably produced pioneering customer product with CO_2_ as low as 300 kg/tonne (3) ‘XCarb`™ innovation fund for breakthrough technologies for net zero steelmaking”. The company indicated to produce 600,000 tonnes of equivalent green steel available by the end of 2022 (climate change, carbon dioxide emission; ArcelorMittal^ 2021^).

Peak 6, 06.05.2021: Most of the media posts related to a financial statement by ArcelorMittal. The company reported a net income of USD 2285 million for the first quarter 2021 compared to a net loss of USD 1120 million for the first quarter of 2020. The first quarter of 2021 was the strongest financial result in a decade (litigation/legal issues; The Indian Express 2021).

#### Incident monitoring (June 2019 to June 2021)

The three strongest incident risk categories were regulatory/advisory, labour issues and ground transportation problems. Most incidents for the tracked mines were reported for Australia, Brazil and South Africa (50.6% share of global production) (Table 3). The incidents include a large number of reports related to the COVID-19 pandemic in 2020. For example, several media posts related to border closures between January and April 2020 authorised in Canada, South Africa, Brazil and Australia. For the studied period, all larger mines in the Brazilian Iron Quadrangle (5.9%) and some in other countries were directly affected by COVID-19 at the beginning of the pandemic, others through shipping problems at ports in Western Australia and Brazil or environmental risks (< 0.1 to 2.3%, highest Area C, Table 3).

#### Selected severe and moderate incidents

#### Australia

##### *Selected COVID-19-related incidents*

*Regulatory advisory (severe)*: In March 2020, the Australian government implemented strong COVID-19 measures to counteract the spread of the disease. On March 28, social distancing measures were implemented for a minimum of 6 months, including quarantine enforcement for all arrivals at airports nationwide (28.03.2020, Australian Dpt. Of Health, 2020). To date, Australia enforces strong COVID-19 measures including limited access for residents and non-residents entering the country as well as travel restrictions between Australian states (Australian Government Department of Home Affairs, 2021).

##### *Other incidents*

*Security, weather, storm, infrastructure, power outage (moderate)*: Australia’s Bureau of Meteorology issued on 23 March, 2020, severe weather warnings in every state and territory nationwide, impacting around 10 million people. The authority also issued evacuation orders due to heavy rainfall and flash flooding of rivers, main roads and bridges across multiple areas of the country. Coal deliveries to the Port of Newcastle (north of Sydney, world’s biggest coal export port), essential for steel making, were halted because of rail line floodings (The Washington Post, 2020). In May, ex-tropical storm Mangga led to infrastructural and power grid damages as well as power outage of about 50,000 households in Western Australia. Strong winds raised dust through large areas including Geraldton (DHL Resilience360, 2021). Geraldton also has an important terminal for iron ore shipments (Mid West Ports Authority, 2020).

##### *Cyber attack/ground transportation (moderate)*:

On June 19, 2020, Australian Prime Minister Scott Morrison stated that the Australian government had been targeted by a “sophisticated state-based actor” cyber attack for months, including all levels, essential service providers, other operators of critical infrastructure and political organisations. He recommended that businesses and organisations should update web or email services and use multi-factor authentication (BBC 2020b).

#### Brazil

##### *Selected COVID-19-related incidents*

*Health/outbreak of disease, strike, civil unrest/protest (severe to moderate)*: In March 2020, Brazil implemented various measures to counteract the COVID-19 pandemic. On 19 March, the Brazilian government announced an immediate closure of the country’s borders for at least 15 days, excluding the border to Uruguay. Entry bans for travellers from European and Asian countries came into force on 23 March lasting at least 30 days. Various other measures continued (health/outbreak of disease; A3M Global Monitoring, 2020). However, towards 2021 Brazil is number three regarding total infections after the USA and India and number two regarding death tolls after the USA. COVID-19 also affected several other services. In 2021, GardaWorld reported on May 26, 2021, that anti-government protests would occur in more than 85 cities nationwide on 29 May. Activist groups including “Popular Brazil” and the “Black Coalition for Rights” would hold demonstrations to condemn the government's handling of COVID-19 and demand the impeachment of President Jair Bolsonaro (civil unrest, protest/riot, moderate; GardaWorld 2021).The demonstrations were to take place in various cities including Belo Horizonte, the centre of the Brazilian “Iron Quadrangle”.

*Production halt (severe):* At mine level, Vale S.A., for example, had ordered the closure of a series of mines at Itabira in Minas Gerais state of the “Iron Quadrangle” on June 6, 2020, due to COVID-19 concerns, after 188 people tested positive and the Minas Gerais Labour Court had ordered the suspension of mining. More than 10 percent of the company’s iron ore output was estimated to be offline, possibly impacting global iron ore markets. (Reuters 2020i).

##### *Other incidents*

*Health, outbreak of disease (non-*COVID-19*; moderate)*: Over 332,000 recorded cases of dengue fever across Brazil since the beginning of 2020, including up to 77 related deaths, were reported on March 25, 2020, an increase of 45% compared to the same time period in 2019 (DHL Resilience360, 2021). Further reports indicate a spread of the disease in important iron ore mining states such as Minas Gerais, Pará and Mato Grosso (Travelvax 2020).

*Production halt, cargo disruption, port closure (moderate):* On April 16, 2021, media reported that Companhia Siderúrgica Nacional (CSN) was fined by local authorities for allegedly dumping industrial waste into the ocean without adequate monitoring. In consequence, the company was pushed to suspend their iron ore and container terminal operations in the Port of Itaguai. CSN operates two terminals: the Tecar bulk solid terminal with a capacity of 45 million mt/year of iron ore (ca. 1.9% share of global iron ore production) and the Sepetiba Tecon container terminal. CSN in total is ranked Brazil’s second-largest iron ore exporter (DRYAD Global 2020). On June 4, 2021, Vale S.A. had to stop its Mariana mine complex near its Xingu dam following a safety notification from regional authorities. The company stated that the Xingu Dam was at level 2 of the “Emergency Action Plan for Mining Dams”, in which there would be “no imminent risk” of rupture. The closure had an estimated impact of 33,000 tonnes per day of iron ore production (Production halt; Reuters 2021l). The shutdown Xingu tailings dam in the town of Mariana already devastated the region by a dam rupture in 2015 which killed 19 people. The second dam rupture occurred in 2019 at B1 dam in Brumadinho, Minas Gerais state, which killed 270 people. A regional labour judge ordered Brazilian miner Vale SA on June 10 to pay 1 million reais (US$ 197,240) in compensation to each of the families of the 131 employees which were killed in the dam failure (Reuters 2021k).

#### Venezuela 

##### *Selected COVID-19-related incidents*

*Health, outbreak of disease, regulatory advisory (severe)*: On March 13, 2020, President Maduro announced that all flights from Europe and Colombia will be suspended for 30 days (Argus Media, 2020). On March 16, Venezuelan Vice President had announced that schools and public gatherings will be suspended starting on March 16. On April 20, local government reported that they will halt all commercial activities as well as public transport in the Caroni municipality of the state of Bolivar from April 26 to counteract the spread of the coronavirus. It was not immediately clear how long these measures would remain in place (DHL Resilience360 2020). The iron ore mine Ferrominera Orinoco operates in the State of Bolivar, which at that time could have been affected by the restrictions.

##### *Other incidents*

*Fuel disruption, trade restrictions, regulatory, customs regulation (severe)*: Fuel shortages and hour-long queues across Venezuela over weeks were reported for March and April 2020 at petrol stations. The shortages were said to be an effect from government mismanagement and corruption as well as economic collapse on top of the COVID-19 pandemic (CSIS 2020). On April 22, 2020, US administration ordered Chevron Corporation and other oil and oil service companies to “wind down” operations in Venezuela by December 1, including drilling for oil or exporting oil to increase the pressure on President Maduro to step down (AP News, 2020). On April 25, 2020, US oilfield services provider Halliburton Corporation stated that it would cease most of its operations in Venezuela following the US decision (regulatory and trade restrictions, severe; DHL Resilience360^ 2020^). As of June 29, 2020, a new US regulation impacted shipments from the USA to China, Russia and Venezuela, changing the licencing requirements for exports, re-exports and in-country transfer of certain items, export declaration requirements for these countries regardless of value. The new regulation also required the correct “Export Control Classification Number (ECCN)” or EAR99 indicator, per line item, in all export declarations (regulatory, trade restriction, customs regulation, moderate: Konkel and Consultant 2020).

*Civil unrest, infrastructure, fuel disruption, power outage, protests (moderate)*: Due to the lack of fuel and natural gas ongoing nationwide power cuts were reported. The capital and other major metropolitan areas such as Maracaibo, Maturin, Valencia, Barquisimeto, Ciudad Bolivar and Guayana City were without electrical service for over 14 days. Protests were also reported (DHL Resilience360 2020).

#### South Africa

##### *Selected COVID-19-related incidents*

*Ground transportation, border closure/delay (severe)*: South Africa closed all land borders starting January 11 until February 15. Exceptions were made for the transportation of fuel, cargo and goods and emergency medical (CNN 2021b).

*Regulatory advisory, maritime advisory, port disruption, port closure (severe)*: On March 23, 2020, South Africa implemented a nationwide lockdown scheduled until May 1 to prevent the spread of COVID-19. As a critical supplier of raw materials, it was announced that the pandemic was likely to impact supply chains across the world. On March 18, government authorities already banned all vessel crew changes at South African ports. Some passenger terminals but no cargo terminals were closed. Vessels would be cleared only after checks by the Port Health and Transnet National Ports Authority. On March 24, Transnet reported that it would finally close its bulk terminals from March 26 until on April 17. Essential commodities such as grains, soy, fertiliser and wood chips were exempt (Fastmarkets 2020; Shipping and Freight Resource 2020; Everstream Analytics 2020). Transnet operates an iron ore terminal at Saldanha port (Transnet, 2021).

##### *Other incidents*

*Infrastructure, power outage (moderate)*: On March 10 2020, the state power company Eskom Holdings SOC Ltd. doubled the severity of nationwide power cuts. The company implemented “Stage 4 outages” for its nuclear plant Koeberg, removing 4000 megawatts from the plant, after an additional breakdown of 930 megawatts from the Koeberg Unit 1 (Bloomberg 2020).

### Platinum and palladium

### Overview and country risk ranking

Platinum group metals (PGMs) are mainly used for autocatalyst (61%), jewellery, electrical applications and chemicals (total 89%) and other applications (IPA, 2021). Ninety-eight percent of the supply of PGMs is from only five mining countries. The three largest mining countries are South Africa, Russia and Zimbabwe (total 88.3%, DERA, 2021b). For this study, 21 mines for platinum covering 97.5% of global production and 20 mines for Palladium covering 89% of global production were analysed (S&P Global Market Intelligence, 2020b, Data 2018) (Table 1). Platinum and palladium occur in mineral paragenesis and are mined together. However, the PGM distribution is different in various regions. The five largest Platinum mines worldwide are Rustenburg, Marikana, Impala and Mogalakwena mines (South Africa) and Stillwater (USA). The five largest Palladium mines/regions worldwide are the Kola Division (Russia), Polar Division (Russia), Mogalakwena, Impala and Marikana mines (South Africa).

In general, the market concentration for PGMs at country level is high (HHI: Pt = 5402; Pd = 3178), and the weighted country risk is moderate (WGI: Pt = 0.06; Pd =  − 0.04) (DERA, 2021a). The overall exposure to country risk is rather high (average: Pt = 40.7; Pd = 40.7) (Table 1). The best-performing socio-political frameworks in which PGE mines operate are located in Canada, namely Sudbury, Lac des Iles, Raglan, Ontario Division and Sudbury Operations (risk ranking < 30). Stillwater mine (USA), the mines in Russia and about half of the mines in South Africa operate under an overall risk exposure between 30 and 50, all other PGE mines in South Africa as well as the PGE mines in Zimbabwe are exposed to an overall exposure to risk of > 50. Several mines operate in an environment with a > 70 risk ranking in certain risk categories: Mines in South Africa for operational risk plus high risk for labour strike, civil unrest and extreme weather events. Selected mines are exposed, for example, to flash floods or hail. Mines in Zimbabwe for socio-political and sustainability issues, corruption, customs inefficiency, detention and discrimination, weak law enforcement and worker’s rights and personal freedom. Mines in Russia are highly exposed to social and political risk, corruption, customs inefficiency, weak law enforcement and some extreme weather events.

### Web scraping results for platinum (January to June 2021)

The highest number of media posts falls in three risk categories: mergers and acquisitions, litigation/legal issues, incidents/accidents (30.0%). In general, each major peak results from one or multiple incidents. For platinum mining, five peaks are prominent (ca. >  = 300 posts in one day, various languages, each peak covers various risk categories). The posts mainly cover reports about activities by Glencore, Sibanye-Stillwater and Nornickel (Table 1). Based on the observed incidents, the highest peak (Peak 1) correlates with Nornickel’s payment of fine of US$ 1.99 billion; otherwise, no effect on the price was found (Fig. 6d).

Peak 1, 15.02.2021: Most of the posts covered multi metal miner Glencore, which also operates the Raglan nickel-platinum-palladium mine in northern Quebec, Canada (90%). Glencore reported US$ 3.3 billion in earnings before interest and tax at its trading business and core earnings for the full year 2020 were $11.6 billion EBITDA. However, the turnover slumped 34% to US$ 142.34 billion (Glencore, 2021a, b).

In Russia, Vladimir Potanin achieved a new Russian wealth record of US$30 billion despite his 35% owned mining giant Norilsk Nickel/Nornickel had to pay US$ 1.99 billion fine (146.18 billion rubles) over an Arctic fuel spill at Norilsk (Forbes 2021). Company shares rose by 40 percent in 2020 as a result of a sharp increase in prices for nickel, copper and palladium. The Russian company is the world’s largest producer of palladium and one of the largest of nickel.

Peak 2, 10.06.2021: Over 70% of the posts dealt with the death of two miners at Stillwater mine in Montana, USA (owned by South Africa-based Sibanye-Stillwater). The two miners died in an underground accident (Montana Standard, 2021). The workers drove a utility vehicle which crashed into an underground locomotive.

A larger number of posts covered news about iron ore miner Vale, who also operates nickel-platinum-palladium mines in the Sudbury basin of Ontario, Canada. According to Brazilian authorities one of Vale´s iron ore tailings dams, Xingu dam, would be at risk (see also section “iron ore”).

Peak 3, 02.03.2021: Over 90% of the posts covered a report by the Aboriginal Areas Protection Authority in Australia, which stated that Glencore, also a PGE miner, came close to a culturally significant Aboriginal area at the McArthur River lead–zinc mine (see also section “Zinc”, Peak 1). Other news pointed to an accusation by the former Eskom company chief executive Brian Molefe who accused South Africa’s president Ramaphosa of using his political influence to favour Glencore in a coal deal, at a time when he was Optimum Coal Holdings shareholder and chairperson, owned by Glencore (SABC News, 2021a, b).

Peak 4, 04./05.02.2021: Various news about Glencore were published on February 5, not directly in connection with the platinum business. Glencore and US-based Century Aluminium Company agreed to deliver 150,000 tonnes of “Natural Aluminum” (produced from 100% renewable energy) to Austrian company Hammerer Aluminium Industries (HAI) (Glencore, 2021a, b). Other posts about Glencore were related to a former state acquisition of Glencore’s (and Vedanta’s) local copper mine operations in Zambia 2 years ago. On February 2, Zambian government officials indicated that they were not looking “at any other specific mining operations that we want to go into a relationship with”, following a statement by the president of Zambia about taking over other mining firms by force or nationalisation (Mining.com, 2021a, b).

Further news were published about Vale, which operates the Nickel-PGE Subury operations in Canada (see iron ore: environmental spill at Vale’s Brumadinho mine in Brazil).

A larger number of media news on February 5 report about the enormous profit of PGE miner Sibanye-Stillwater despite COVID-19 disruptions in 2020 (Mining Weekly 2021). Sibanye, which operates gold and PGM mines in South Africa and PGM mines in Zimbabwe and the USA, advised their shareholders to expect attributable profit for the year to end-December of between Rand 28.7 bn and 29.9 bn.

In Russia, judges at the Krasnoyarsk Court of Arbitration announced that Norilsko-Taymyrsky Energy Company, a subsidiary of the PGE miner Norilsk Nickel, had to pay a fine for environmental damage caused by a massive fuel spill in the Russian Arctic in 2020 (see also section “Copper”).

Peak 5, 17.3.-19.03.2021: Peak 5 mainly consists of news about two PGE mining companies, Sibanye-Stillwater and Glencore. Siybanye-Stillwater formed a partnership with Johnson Matthey, a major semi-fabricator of PGEs, to work together to achieve and improve ESG (Environmental, Social and Governance) standards (Johnson Matthey, 2021). The two companies also extended their PGM supply and refining agreement to ensure a long-term sustainable supply. Other news covered information to shareholders about specific performance conditions of award payments of conditional shares in March 2018 under the Sibanye-Stillwater Share Plan 2017 (Sibanye-Stillwater, 2021).

Glencore was in the headlines because the Australian RMIT Business and Human Rights Centre accused Glencore to have failed to adequately obtain consent from traditional land owners prior to acquire land rights (RMIT University Australia, 2021).

### Web scraping results for Palladium (January to June 2021)

The highest number of media posts falls in three risk categories: Governance, Labour practice and incident/disruption (61%). In general, each major peak results from one or multiple incidents. For platinum mining, five peaks are prominent (ca. > 15 posts in one day, various languages). The posts mainly cover reports about activities at Lac des Iles, Unki/Zimplats and East Boulder mines (Table 1). Based on the observed incidents, no effect on the price was found (Fig. 6e).

Peak 1, 08.03.2021: Most of the media posts cover activities in the area of the PGE Lac des Iles Mine near Thunder Bay, Ontario, Canada, owned by Impala Platinum Holdings. In that area, another competitor, Clean Air Metals, announced that it had filed a NI 43–101 technical report detailing the mineral resource estimate for the Thunder Bay North Project, a platinum, palladium, copper, nickel project located near the City of Thunder Bay (Clean Air Metals, 2021).

In Zimbabwe, a new government regulation compelled exporters and miners such as Anglo American Platinum and Zimplats, which operates the Unki and Ngezi platinum-palladium mines, respectively, to sell 40% (previous 30%) of their foreign currency export earnings to the Reserve Bank of Zimbabwe. The Chamber of Mines of Zimbabwe reported that it started talks with the government to annul these regulations (Zimbabwe Situation, 2021).

Peak 2, 14.-15.02.2021: Peak 2 mainly consists of news about the companies Sibanye-Stillwater and Zimplats. Sibanye-Stillwater announced that the group’s mineral resources and mineral reserves as at December 31, 2020, increased 40% in PGM primarily due to the takeover of entire share capital of Lonmin (Bloomberg, 2021). The Lonmin assets included the Marikana K4 + project, the Klipfontein opencast project as well as PGM mining operations and associated retreatment, smelter, base metal refinery and precious metal refinery assets in South Africa.

At Zimplats, a mine accident occurred on February 15, 2021. An employee of one of Zimplats contractors was injured and the death confirmed when a high wall collapsed at the entrance of Ngwarati Mine in Mhondoro-Ngezi, Zimbabwe (The Herald, 2021).

### Incident monitoring (platinum and palladium, June 2019 to June 2021)

Reported severe and moderate incidents for PGEs include a large number of reports related to the COVID-19 pandemic in 2020 plus other incidents. Most severe incidents fell in the risk categories regulatory advisory, labour and ground transportation/border closures (Table 3). Extreme incidents did not occur. Most incidents for the tracked platinum and palladium mines were reported for South Africa, Canada and the USA (43–88% share of global production, nationwide and local incidents). Severe incidents include a large number of reports related to the COVID-19 pandemic in 2020. For example, production halts in South Africa occurred from March 2020 as well as curfews and other restrictions due to the COVID-19 pandemic. For the studied period, all larger platinum and palladium mines in South Africa (71% and 26% share of global production, respectively) were directly affected by production halts at the beginning of the COVID-19 pandemic at mine level, in particular at Anglo American Platinum, Sibanye-Stillwater and Impala Platinum mines (Table 3). A few other larger mines were potentially affected by other incidents, e.g. by civil unrest in the Rustenburg area in South Africa (ca.40% share of global production for platinum and 12.3% for palladium) and at Nornickel in Russia (Polar Division, 14.7% share of global production for palladium and 4.2% for platinum) due to an environmental spill, mostly for a very short period.

#### Selected severe and moderate incidents

#### South Africa

##### *Selected COVID-19-related incidents*

*Ground transportation, border closure/delay (severe):* South Africa closed all land borders starting January 11 until February 15. Exceptions applied to the transportation of fuel, cargo and goods and emergency medical (CNN, 12.01.2021).

*Production halt, force majeure, health, outbreak of disease (severe and moderate)*: Anglo American Platinum and the PGE producers Sibanye-Stillwater and Impala Platinum declared force majeure on supply from South African mines on March 30, 2020, following the national lockdown of March 26 to slow down the spread of COVID-19 coronavirus infections (severe, Reuters 2020k). On March 27, Tharisa Mining announced a halt on all operations at its mines with effect of March 26 (severe, Fastmarkets 2020b). On April 1st, Northam Platinum declared force majeure to its customers on PGM supply. Northam Platinum reduced capacity to one furnace and considered limited mining at its Booysendal mine due to COVID-19 measures (Business Day 2020a). Following in May 16, Impala Platinum announced that it would temporarily close the operations at Marula mine due to COVID-19 infections among workers. The mine stopped operations on May 17 (moderate, Reuters 2020j).

##### *Other incidents*

*Production halt, force majeure (severe and moderate)*: After an explosion on February 10 at the Anglo Converter Plant (ACP) “Phase A Plant” at Waterval smelter in Rustenburg, Anglo American Platinum declared force majeure on delivery of PGEs on March 6, 2020. On March 6, Anglo American Platinum reported that water was detected in “Phase B Furnace” unit, which posed a high risk of another explosion. Repairs to fix the “Phase B Furnace” unit would take about 80 days (severe, Anglo American 2020). Further problems at Anglo American Platinum on November 6, 2020, led to the closure of the ACP “Phase B Furnace” unit due to water leaks. The closure was a measure to ensure environmental safety. The company lowered its refined production and sales by around 20 percent (moderate, Anglo American 2020).

*Civil unrest, protest/riot, non-industrial fire, power outage, public safety/security (moderate)*: In 2020, production at several PGE mines in South Africa were at risk due to several regional and nationwide protests and strikes. For example, social unrest due to power cuts were reported in Rustenburg on June 4, one of the centres of PGE mining in South Africa. Protesters burned tires, damaged houses and vehicles and set fire to electrical boxes (The South African 2020). On October 7, 2020, protests and strikes were expected to occur in towns and cities across the country. The Congress of South African Trade Unions (Cosatu) and the SA Federation of Trade Unions (Saftu) announced to demonstrate against state capture, corruption and gender-based violence. Cosatu and Saftu stated that they would cause “as much disruption as possible through these demonstrations” (Africa News 2020). On November 2, the National Education, Health and Allied Worker’s Union (Nehawu) announced a nationwide strike for November 26, 2020, in response to the government´s decision to freeze public-sector wages (Businesstech 2020).

*Regulatory, trade restrictions (moderate)*: Government authorities approved an export tax on chrome ore to support the domestic ferrochrome industry. In some mines, PGEs are produced as a byproduct of chromite, e.g. in chromite mines held by Tharisa and Jubilee Metals (Reuters 2020l). The new export tax could have had a side effect on PGE production.

##### Zimbabwe

###### *Selected COVID-19-related incidents*

PGE production in Zimbabwe was not interrupted by national COVID-19 measures in 2020. Anglo American Platinum Unki mine and Impala Platinum Great Dyke mines were running at full capacity (Business Day 2020b). All Zimbabwe’s platinum mines received government dispensation to operate during the country’s lockdown.

###### *Other incidents*

*Cargo, civil unrest, cargo disruption, protest/riot (severe):* Security measures on July 30, 2020, by government authorities particularly in the Bulawayo and Harare were introduced after a popular national politician was suspected to have been poisoned on July 29. Major protests were expected for July 31. Heavy roadway security denying passage was implemented. As a result, freight movement in these regions and other areas could have been severely disrupted and civil unrest and security measures were expected to follow (DHL Resilience360 2020).

*Labour, general strike (moderate)*: The Zimbabwe Congress of Trade Unions planned a general strike on September 23, 2020, to protest against poor working conditions and corruption, as well as salaries to be paid in US dollar currency. The planned protest followed workers protest every Monday from August 31 near their workplaces (DHL Resilience360 2020).

##### Russia

###### *Selected COVID-19-related incidents*

In general, Russian PGE producer Nornickel announced on April 30, 2020, that “…all production assets of the Company operate in a business-as-usual mode while dedicated emergency response teams continuously monitor the situation on sites” (Bloomberg 2020).

*Ground transportation, border closure/delay, cargo, postal disruption (moderate)*: On March 30, all land borders of Russia closed due to COVID-19 measures, at least for a period until May 1st (07.04.2020). COVID-19-related measures impacted, for example, postal operations in Russia. On March 26, Russian Post indicated that it was unable to guarantee compliance with delivery standards. As a result, Russian Post was considering a situation of force majeure concerning the quality of service and remuneration for all categories of mail (DHL Resilience360 2020).

###### *Other incidents*

*Production halt, chemical spill, regulatory advisory, environmental regulations (severe)*: On May 29, 2020, a 21,000 L diesel fuel spill occurred at Nornickel´s Heat and Power Plant № 3 (HPP-3) in the Kayerkan neighbourhood of the city of Norilsk (Nornickel 2020). On July 7, 2020, Nornickel was requested by Russian environmental organisation Rosprirodnadzor to pay RUB 148 billion (EUR 1.84 billion) for these damages (Reuters 2020m).

*Civil unrest, protest, riot (moderate)*: Alexei Navalny called for nationwide protest after a judge ruled that he should remain in custody for violating terms of a suspended jail sentence. On January 18, 2021, at least 70 supporters and journalists were arrested. Rallies were expected for 23 January to demand his release. A large police presence and further arrests were expected (Reuters 2021m).

## Discussion

Commercial supply chain monitoring tools were originally developed to track a certain product/good along the value chain to its destination. From a mineral economist, investor, analyst or strategic buyer perspective, tracking a whole market segment, including all important supplies for mineral raw materials in many locations and regions in the world is of great interest, too. Thus, this paper selected the most important mines and company headquarters for each studied mineral raw material and observed the information flow for geographic locations of mines and for mining company headquarters. The major aim was to analyse relevant risks and their impact on supply. The major difference to commonly available risk assessments and rankings for mineral raw materials is that—using big data in combination with artificial intelligence—risk information is provided: (i) in near real-time; (ii) it includes short term risks; and (iii) may have predictive character of forthcoming disruptions of the supply chain. Thus, the reaction time for companies is early and precise according to specific risks. Most of the current criticality analyses refer to medium to long term risks, e.g. the general market concentration of production (HHI, see below), supply/demand estimations, substitution and recycling options, etc. (see, e.g. Rosenau-Tornow et al., 2009; DERA, 2021a, b; European Commission, 2022; British Geological Survey, 2022; U.S. Geological Survey, 2022). The medium- to long-term risks certainly provide an assessed and profound background as to how volatile a market is over a year or 2; however, they fail to take account of short-term incidents. These, on the other hand, may also have a profound adverse impact on supply chains and markets. Hence, the adoption of big data analytics narrows the information gap existing in many raw material markets.

For this study, we used a big data tool provided by Everstream Analytics. It implements a big data approach by means of the variety of data sources and processing techniques described in Section 3. Together, they account for 1 million data sources in 25 languages that increase the transparency of the supply chain and their fragmented tiers. In comparison, Reslinc digests more than 1.7 billion news feeds from about 3.5 million sources in more than 100 languages, which is an advantage to access primary language sources (Resilinc 2020). Prewave (2021) puts forward to process more than 100 countries in over 50 languages. They also use comprehensive advanced artificial intelligence technology to identify, track and rate risk incidents in an automated process. All approaches also include predictive analytics, which is getting increasing attention in all supply chain risk management solutions. The trend of these systems is to turn the dominant nowcasting into forecasting to allow more time for reaction. Prewave, for instance, was able to determine a strike of 97 seaports across Indonesia by catching up social media messages at grassroot level. Prewave was able to analyse the messages with machine learning technology and predicted the strike alert as long as 14 days in advance. The exact date of the strike was predicted 5 days in advance (Prewave 2017). The showcased example has important implications how other forms of media, especially the internet and social media, may help to increase the resilience of the supply chain turning information into competitive and financial advantage.

The differences and advantages of each approach need to be investigated in more detail in future. It becomes clear that the current amount of data can only be processed with a large degree of automation. Further scientific work about how to adjust of such big data and artificial intelligence tools to certain research questions is necessary to verify their effectiveness.

In principle the study shows that the applied methods are very promising approaches to track at least some potential market risks: (i) ranking the country risk using socio-political, environmental and natural occurrences frameworks in which mines operate based on annual data, (ii) web scraping of media posts and (iii) incident monitoring based on ranking of the severity of incidents in near real time. The web scraping results were additionally compared with price data for mineral raw materials to test whether certain events had an immediate effect on the price. It is highly recommended to improve the design of search algorithms through artificial and machine learning techniques especially for web scraping. Furthermore, we experienced that the indicators being monitored need to be adjusted for each studied mineral raw material depending on what question should be answered, whether it be supply risks, price risks or key performance indications for suppliers.

It certainly would be of great interest to further investigate risks for intermediate products of the higher value chain, which are manufactured from the primary mineral raw materials. However, this was not focus of the current study. For future work, it would be necessary to compile databases of major global producers of intermediate products first, which could then be tracked in more detail. To our knowledge and for a wide range of intermediate products, no such database exists.

### HHI and country risk ranking

The HHI normally measures the market concentration of companies in a certain market segment and indirectly points to the vulnerability of a market. A global market with only few suppliers will react more sensitive to risks than a market with a large supplier base. Among the 12 mineral raw materials studied, platinum mine production has the highest global market concentration. For example, COVID-19 measures in South Africa, which is an important PGE producer, potentially had a higher impact on the raw materials market than measures in Peru and Chile. Peru and Chile both are important copper producers, but the copper market is more diversified, thus the impact on the raw materials market is lower (72% share of global PGE production potentially affected by measures in South Africa, compared to 27% share of global copper production potentially affected by measures in Peru and Chile, Table 1). The HHI is a good indicator to decide, which markets should be monitored more closely compared to markets, which are more diversified (Rosenau-Tornow et al., 2009; DERA, 2021a, b; European Commission, 2022; British Geological Survey, 2022; U.S. Geological Survey, 2022).

The results for the country risk rankings for the 12 studied mineral raw materials show that the overall country risk exposure of mines is moderate. However, the results also show that mining companies operate in more risky socio-political frameworks with regard to corruption and law enforcement. Country risk rankings may be a first indication for potential supply risks or they may point to a more difficult socio-political framework in which mining companies operate. However, they do not rate the performance of a company and their mine sites. A good example is the comparison between lithium and cobalt mines: the HHI for lithium and cobalt is high, but the country risk rankings according to the World Bank are different. Lithium is mined in countries with a low country risk (WGI: 1.16), whereas cobalt is mined in countries with a high country risk, mainly due to mining in the DR Congo, which is by far the largest cobalt producer (WGI: − 1.15) (DERA, 2021a). Similarly, according to Everstream Analytics, the country risks for cobalt are much higher than for lithium (value for lithium and cobalt: operational risk 40:47; risk for political violence 23:37; risk to individuals 29:56; socio-political risk 46:63; sustainability risk 42:63).

Continuous monitoring of the HHI and the country risk may show how the risk rankings may develop over time and what the implications to achieve a more secure and sustainable supply chain may be.

### Web scraping

Over a period of 6 months and for all 12 mineral raw materials, the number of media posts was as high as 1400 posts in one day. The number of media posts for mining company headquarters was a lot higher than just for mine locations (Figs. 3A and B). Some company headquarters popped up more frequently in the analysis, for example Glencore, BHP Group and Rio Tinto. Incidents or reports about these global mining giants are more frequent than those for smaller or less diversified mining companies. However, in highly concentrated markets, where a few small mining companies dominate a market, incidents affecting company headquarters or mines could become highly relevant. For example, SQM and Albermarle are—compared to Glencore or BHP Group—smaller mining companies, but they are strategic to the global lithium market. By far the largest graphite producer outside China is Syrah Resources, which operates Balama mine in Mocambique. Like SQM and Albermarle for lithium, Syrah Resources is a relatively small company but owns a large share of global graphite production. Another example are government owned assets, for example, the State of Guinea, which operates Sangaredi bauxite mine together with a consortium of larger aluminium producers (La Compagnie des bauxites de Guinée, CBG). Although a minority shareholder (49% share of assets), political instability in the country such as the military coup in September 2021 could have harmed the whole bauxite operation, keeping in mind that Sangaredi is the fifth largest bauxite mine globally and Guinea the third largest bauxite producer in the world. Web scraping of these markets would immediately point to potential critical situations, if such companies would be in trouble. The results of the web scraping exercise pointed to other interesting developments, for example, to social risks at McArthur River zinc mine or to financial burdens due to environmental disasters at mines.

In some cases, the web scraping methodology led to false results or conclusions. For example, web scraping of the biggest copper mine El Teniente in Chile resulted in media posts about certain activities of a Chilean lieutenant (teniente being the Spanish term for lieutenant). Other wrong media assignments to search results were related to the Grasberg copper mine in Indonesia (“Grasberger Bauausschuss”, translates to Grasberg building committee in German), a fire outbreak in the USA at “Copper Hill road” in Colorado state, or for an incident at TX-based oil firm “Copper Ridge Resources” (keyword search “copper”). Improved artificial intelligence would help to create better results and reduce manual efforts to check data. Web scraping results based on the name of mining company headquarters often led to media posts, which were not relevant to the mineral raw material under investigation. For example, media posts for Newman iron ore mine, operated by the BHP Group, included incidents at Escondida and Spence copper mines in Chile, because they both are operated by the BHP Group. Incidents for headquarters thus need to be interpreted in a different context. They are useful if certain incidents may jeopardise the financial stability or environmental and social performance of a company. Checking the creditworthiness of mining companies through rating companies could also be a useful instrument.

### Web scraping results and raw material prices

A simple visual comparison between the web scraping results and price data for the five studied mineral raw materials did not reveal any good visual correlation. Only in two cases, a price shift was observed: one for copper and one for platinum. The increase in the copper price was directly related to an incident at Las Bambas mine in Peru (Peak 1, 05.01.2021). The incident, a road blockage, hindered the export of copper concentrates over weeks. However, this incident was not the only reason for the price shift. Instead, it was a combination of several factors: declining warehouse inventories at the London Metal Exchange and the Shanghai Futures Exchange combined with potential supply gaps from other copper mines in Peru (and possibly other countries) due to strict COVID-19 measures and mine closures all over the country. The increase in the platinum price (Peak 1, 15.02.2021) might have been supported, not only, by Nornickel´s fine of US$ 1.99 billion, which a Russian court imposed on an environmental disaster at Norilsk.

In 2020, the COVID-19 pandemic had a clear impact to almost all mineral raw material prices between January to April 2020 and at the beginning of the second COVID-19 wave in September/October 2020, although the price decrease was not related to a single event or incident. During the first COVID-19 wave, global growth decreased dramatically, and consequently global demand for mineral raw materials and their prices decreased (DERA, 2020).

In principle, certain incidents may have a strong effect on price, especially in highly concentrated markets. For example, cobalt prices peaked due to a supply shock during the first and second “Congo wars” in Zaire and the DRC in the 1990s/early 2000s, because the DRC was the biggest cobalt producer in the world, and still is today (Buchholz et al., 2020, and references therein). Any instability in the DRC would immediately have an impact on the cobalt price, even today. Sometimes, prices move upwards or downwards with a time lag. For example, real prices for tin significantly decreased in 1986 after the International Tin Cartel finally collapsed in 1985. A risk event, which impacts prices at an early stage, could actually increase the prices within days or weeks, if the event, e.g. a force major or strike, lasts for a longer period of time and affects the global market. Web scraping in combination with price monitoring may thus have early warning properties.

Wanner et al. (2014, and references therein) studied determinants of the price for the high-tech metals neodymium, indium and gallium in an “event study” using a dataset of prices and news items. They were able to detect coinciding events to almost 90% of all price jumps/drops and showed that if certain events occur with a probability of over 50% a price jump within 10 days will follow. According to their experience, further correlation analysis using big data analytics tools in the long run and for a wider range of mineral raw materials would add to a better understanding of price drivers. An analysis like that would also improve available supply risk management tools applied to the mineral raw material markets.

### Incident monitoring based on manual intervention and severity

It seems that certain types of incidents are only relevant to some mineral raw material markets and not to others. For example, the management of bulk terminals at overseas ports is more strategic and incidents more severe to mineral raw material markets, which are shipped in bulk carriers like iron ore, bauxite or manganese ore, than for containerised freight such as for minor metals or industrial minerals like graphite. Port disruptions for container ports normally are less severe, because they are more abundant in one country or region compared to bulk terminals.

Certain incidents may have a limited impact on a market, because they only have a short time effect or are otherwise less severe. For example, a mine worker’s strike in Chile or Peru might last for a week or 2, with no relevant impact to the supply chain. But the risk for major supply chain disruptions and social governance issues increases the higher the frequency and the more severe these strikes become. Thus, the incident type and the severity of a specific market need to be adjusted to individual markets.

Human intervention in data processing is a natural source of error. Ranking the severity is based on manual intervention and expert rating. In rare occasions it was found that the same media post about an incident was rated moderate in one media post and severe in another.

#### Supplier risks

COVD-19 disruptions widely affected the mining sector especially at the beginning of the pandemic between January (start in China) and June 2020. As of June 2020, a total of 275 disrupted mining operations in 36 countries and for 13 mineral raw materials were identified worldwide (S&P Global Market Intelligence, 2020b). According to the study by S&P Global Market Intelligence (2020b), Peru, Chile and Mexico were the top three countries with the highest revenue at-risk. However, as shown in our study, most government authorities declared the mining sector as an essential industry and allowed mines to continue operating while implementing protective measures against the spread of COVID-19. For example, mining in Chile went back to relatively normal levels in April 2020. In South Africa, the government allowed companies to reopen mines at 50% capacity, as of April 15, 2020. According to S&P Global Market Intelligence (2020b), only a maximum of 2–3% of global mine production of PGEs and Silver was at risk over a period of a few months; for the other mineral raw materials, it was less than 1% (Table 4, S&P Global Market Intelligence). However, at the beginning of the COVID-19 pandemic, it was not clear how strong the mining sector could be affected by the pandemic. Thus, the risk for production halts was high. According to this study, the highest potential risk for supply disruptions at larger mines due to COVID-19 was in Peru (8.5% of global production for copper; 5.1% for zinc), in the Brazilian “Iron Quadrangle” (5.9% of global production for iron ore) and in South Africa (71% of global production for platinum, 26% for palladium, Table 4). The production halts and force majeure statements by Anglo American Platinum, Sibanye-Stillwater and Impala Platinum were serious incidents for the platinum sector and PGE prices started to rise from May/June 2020.

**Table 4:** Impact of the COVID-19 pandemic in the mining sector (summary of at-risk production for 2020, modified after and with kind permission of S&P Global Market Intelligence, 2020b)

Commodity	Number of mines	At-risk production (April 2)	At-risk production (April 8)	At-risk production (April 16)	At-risk production (April 23)	At-risk production (April 30)	At-risk production (June 18)	2020 global estimated production	Global share of at-risk production (June 18) [%]
Gold (oz)	118	727,996	924,495	1,034,258	1,056,385	1,353,259	1,514,710	107,790,240	1.40
Silver (oz)	101	13,610,744	17,617,841	20,559,719	20,530,777	30,956,910	33,520,249	871,015,860	3.85
Copper (t)	51	224,640	395,405	466,336	466,621	528,470	620,833	20,692,770	3.00
Zinc (t)	24	116,284	113,288	136,731	136,731	199,649	237,606	13,868,240	1.71
Lead (t)	23	26,443	26,034	29,233	29,233	46,636	53,751	5,321,980	1.01
Nickel (t)	16	15,252	12,589	22,772	24,684	24,684	27,599	2,555,910	1.08
Molybdenum (t)	14	2683	6214	7154	7154	8010	8838	331,650	2.66
Palladium (oz)	12	118,050	118,050	115,833	140,191	151,233	151,233	7,072,280	2.14
Platinum (oz)	12	179,261	179,261	174,463	207,515	208,398	208,398	6,286,170	3.10
Cobalt (t)	6	866	637	1057	1057	1057	1170	176,100	0.66
Iron ore (t)	5	3,286,004	3,286,004	6,126,292	6,126,292	6,126,292	7,134,206	2,387,526,390	0.30
Lithium (t)	2	1369	1369	2043	2043	2043	2043	836,930	0.24

Data as of June 23, 2020; Source: S&P Global Market Intelligence

A whole range of further supplier risks were detected during this study, although most incidents lasted for a very short period. For example, power outages potentially threatened PGE mining companies and refineries in South Africa and an explosion at Anglo American Platinum converter plant in March 2020 reduced PGE production output capacity significantly. The market turned even more nervous, when the diesel fuel spill at Norilsk PGE producer Nornickel in May 2020 and strikes in the Rustenburg area in June 2020 occurred questioning a secure PGE supply. Results showed that a series of severe and moderate incidents or a combination of several moderate incidents within a few weeks or months may thus have a serious effect on supply and price. Other examples are a cyber attack in Australia, which corrupted company software nationwide. Initially it was not clear how strong mining companies were hit by the attack, and mine accidents at Ngezi/Implats mine in Zimbabwe or Stillwater mine in the USA in 2021 resulted in legal investigations which could have resulted in interruptions.

#### Socio-political risks

Various socio-political risks were detected during this study. Civil unrest in the form of strikes in Chile, Peru, South Africa and Brazil caused disruptions at or in the vicinity of mines. In June 2020, protestors in Rustenburg area in South Africa burned tires, damaged houses and vehicles and set fire to electrical installations. Follow-up strikes and demonstrations increased the fear for violent conflicts which happened in the past, for example, at Marikana mine in August 2012 when striking mine workers were shot by South African police (South African History Online, 2019). Mines in the Rustenburg area produce around 40% of global platinum and around 12% of global palladium output. Riots in this area are a serious threat to PGE supply.

In Australia, conflicts between indigenous groups and mining companies occurred at Area C iron ore mine and at McArthur River zinc mine close to indigenous sacred sites. Indigenous groups were distressed when iron ore miner Rio Tinto destroyed 46,000-year-old caves at Juukan Gorge in Western Australia subsequent to mine expansion in May 2020 which also caused international criticism. Although Australia has one of the best-performing socio-political frameworks in which the studied mines operate, increasing conflicts between mining companies and indigenous groups in Australia may counteract mine expansion plans or access to new production sites.

Government interventions are another source of risks: In the USA, the trade war with China Government interventions are another source of risks. In the USA, the trade war with China impacted supply chains and the storming of the US Congress by supporters of President Trump impacted ground transport and thus supplies to mining companies. And in March 2021, the Zimbabwean government announced that exporting mining companies would have to sell 40% (previous 30%) of their foreign currency export earnings to the Reserve Bank of Zimbabwe. Escalating inflation hit the country over years, which means that tighter foreign currency control could threaten the whole operation. In June 2021, presidential candidate Pedro Castillo in Peru proposed to raise royalties on mineral sales and had announced tax renegotiation plans in case of his election. Fears over a partial nationalisation of Peru’s mineral wealth were widespread. These cases certainly are severe incidents. However, the risks may not necessarily lead to a supply disruption at a mine, but trade partners need to be aware of the potential risks. More severe trade risks would, for example, be export bans or high export taxes, which—for the five mineral raw materials—were not detected during the period of the study.

A prominent example for political risk is the military coup in Guinea in September 2021, when Colonel Mamady Doumbouya had become Guinea’s interim president (Australian Institute of International Affairs, 2021). Guinea is the second biggest bauxite producer globally; thus, political instability provides high risk for supply disruptions. However, to date, bauxite production and export were not severely affected.

#### Environmental risks

The most prominent environmental damage from mining, analysed in this study, was the dramatic diesel fuel spill at Nornickel Ni-Cu-PGE mine on May 29, 2020, which resulted in legal disputes and outstanding claims for financial compensation. A total of 14.7% of global palladium supply was immediately in focus, at least causing a reputational damage to the company.

Fears of environmental damage caused a production halt at one of the larger iron ore mines, Mariana iron ore mine in Brazil, following a safety notification from regional authorities regarding the nearby Xingu tailings dam (level 2 of the “Emergency Action Plan for Mining Dams, no imminent risk of rupture”). Xingu tailings dam already devastated the region by a dam rupture in 2015 which killed 19 people, followed by a second major dam rupture in Brazil in 2019 at B1 dam at Vale’s Brumadinho mine in Brazil. Mine tailing dams are a serious threat to people and the environment unless professionally monitored. The costs for supervision, monitoring environmental risks and remediation might escalate in future. For example, in Chile, BHP Group announced plans to spend up to $93 million in environmental repairs and reparations following a Chilean lawsuit related to BHP’s Escondida copper mine due to environmental damage in connection with overdrawing water from the Punta Negra salt flat.

Local communities in Australia launched legal action against the Northern Territory government to challenge a decision to lower the McArthur River Mine’s environmental security bond by nearly $120 million. A report by the University of New South Wales (UNSW) and the Environmental Centre of the Northern Territory (ECNT) warned that up to 22 sacred indigenous sites and an important river system would face “irreversible cultural and ecological damage” from the mining operation.

#### Natural occurrence risks

A few natural occurrence risks were found in this study, which however had no major effect on the mining industry. The only extreme incident was a heavy earthquake near Rudna copper mine in Poland in November 2020, which resulted in the evacuation of miners. The earthquake however did not harm any miners or production output. A landslide inside Utah’s Bingham Canyon Copper mine disrupted operations, but mining operations resumed soon afterward with no negative effect on people or the mining output.

Severe and destructive winds and heavy rain throughout Ireland by Storm Ellen were anticipated to cause damages to and flooding of infrastructure, which also could have impacted zinc mines in the country (which did not occur). Heavy flooding in the Tuva Republic in Kazakhstan in 2020 may have caused ground transportation problems for a short while in the area of Kyzyl-Tash Turk zinc-lead-copper mine, but the incident was of no effect to supply. However, seasonal storms in Australia may seriously affect iron ore transport by train or by bulk carriers. Various iron ore terminals at overseas harbours are a bottleneck to global iron ore trade. For example, storms already halted operations twice in the past years at the world’s largest iron ore hub Port Hedland in Western Australia. Port Hedland was closed for 16 h in February 2021 due to Tropical Low 12U, and port Geraldton in Western Australia was affected slightly in May 2020.

A winter storm and cold temperatures in the northern part of Mexico and the US states Texas and New Mexico, which declared force majeure, caused nationwide natural gas shortages and power cuts in Mexico, with minor effect on mines in the country. Although not a major problem for miners, the storm led to the shutdown of manufacturers in the higher value chain for important silicon chip producers in Texas, USA. The event contributed to the severe chip production crisis in 2021 (BBC, 2021).

Natural disaster events could potentially harm mining operations and mine workers seriously, especially in regions known for recurring events. Each mineral raw material should be systematically analysed for such natural occurrence risks.

#### Transportation risks

For this study, only ground and marine transportation risks were considered, and aviation risks were excluded from the analysis. In mineral raw material markets, only high-value materials such as precious metals, precious stones and some minor metals are being transported by air. According to our understanding, potentially affected airports or civil airspace could be circumvented by alternative airports within a country or by cargo transport through neighbouring countries or by using alternative flight routes, so that these risks can rather be neglected. Most of the mineral raw materials are being transported via ground or marine routes. Examples in this study show that ground or marine transportation risks are often connected to other risks types, for example, (i) socio-political risks such as strikes or riots (e.g. road blockage at Las Bambas copper mine, Peru; riots at Marikana PGE mine, South Africa) and (ii) natural disaster risks such as heavy rains and landslides (e.g. road connection to Yauli zinc mine, Peru) or heavy storms and rains affecting key infrastructure like railway lines, harbours and ocean traffic (e.g. iron ore transport in Australia, South Africa and Brazil).

Shipping routes can be at risk, if critical infrastructure such as the Panama or Suez canals is at risk. In 2021, the Suez Canal was blocked for 6 days by the grounded container vessel Ever Given in March following strong winds and difficult manoeuverability of the vessel (nbc News, 2021a, b). The key global trade route came to a standstill disrupting supply chains across industry sectors. Piracy in the Indian Ocean as well as politically motivated potential future conflicts in the South China Sea may hinder maritime transport in future as well. China’s efforts to create a security zone in the South China Sea casts doubt for unhindered access and freedom of navigation in the future (Perthes, 2020). In case of new arctic passages via in the Northern Sea Route (NSR) and the Northwest Passage (NWP), potential geopolitical conflicts and tension may arise between Russia, China and the USA (Gricius, 2021). This may also lead to future transportation problems in the arctic region.

## Limitations of big data analytics

Is big data analytics the new light on the horizon? In many respects, there may be an affirmative response to this question. But there are also pitfalls that prohibit its full implementation. Many data sources are not available free of charge or information on the internet may be data protected. Not all websites are free to be scraped, and it might also be illegal to scrape certain sites by violating their terms of use. Furthermore, automated scraping, storing and analysing data may be a violation against (intellectual) property rights. It should also be stressed that each data source in itself is due to its unstructured nature that is hard to preprocess and analyse, and combining various themes is an even more daunting task. Data integration among different sources is complicated since the data come in various formats. The pooling of data may prove to be very time and resource intense. As an example, raster data from satellites and text mined data from the internet have at first no option to be combined. A merged analysis of both data sources needs further efforts to get the full meaning of the context. Rising data complexity may to this end even impede its widespread introduction. For a broader deployment, standardised communication protocols need to be developed. Increasingly important is also cyber-security. As the internet is still rapidly developing, security attacks get more frequent. There may also be resistance to change against big data analytics since its usefulness may not be seen as better than current decision-making processes.

### Further applications of big data analytics in mineral raw material markets

There certainly are more big data tools available than the ones used in this study. Global Navigation Satellite Systems (GNSS) technology may be used for the observation of all modal types of traffic. Seaborne traffic utilises the Automatic Identification System (AIS). Similarly, air traffic is augmented with the Automatic Dependent Surveillance-Broadcast (ADS-B). Both systems have large analogies and record a vessels or airplanes identification, position, course and speed (also altitude for airplanes and floating depth for vessels) every few seconds having significant benefits for safety and efficiency. The methods could be used to track vessels that transport specific ores like iron ore vessels. The AIS technology has also been used by Stamer (2021) to predict world trade flow using a machine learning approach. Because the information is available in real-time it uses a much more agile approach (Nowcasting) as opposed to traditional forecasting with statistical offices publishing trade data with a lag of several weeks or even months. Augmentation with automatic identification and data capture technologies (AIDC) may track the trade flow of mineral raw materials using small RFID chips or barcodes in containers or vessels similar to luggage tags for luggage control at airports.

Blockchain is a distributed ledger technology (DLT) and reduces time and costs in the entire supply chain. It is also suggested to improve security by tracking the sources of vulnerability in supply chains and in handling crisis situations such as product recalls. It can furthermore help supply chain traceability and transparency and thus facilitate the supervision of responsible production and consumption (Hirata et al. 2020; Burgwinkel 2016, Kersten et al. 2017). Large manufacturers may have 1000 first-tier suppliers, 8000 s-tier suppliers and 30,000 third-tier suppliers (Young et al. 2019, p. 4). BMW, the German car maker, alone has 12,000 suppliers spread out over 70 countries (BMW 2021). Kersten et al. (2020) rightfully assert that especially the blockchain technology is still in its infancy and in many scenarios is not further than the prototype or proof of concept stage. The international trading system in mineral raw materials is a long way from incorporating the full use of the new technology. Nevertheless, information technology can help to shed more light on the more comprehensive supply chain, particularly against the background that the biggest leaps in these technologies are yet to come.

Transparency in the supply chain will also be an increasingly important regulatory requirement. Resources like tantalum, tin, tungsten, cobalt, gold and diamonds may derive from conflict regions. Especially tantalum, cobalt and tungsten are known as critical raw materials; they present supply risks, yet are paramount in technologies for a low-carbon economy (Young et al. 2019, p. 2). Criticality assessments normally still do not delve deeper than the national level, an insight at the firm and supplier level particularly in terms of responsible sourcing in almost all raw materials is missing. But regulatory efforts increasingly shed light on at least the higher-tier supply chain. The smelters and refiners engaging in due diligence programs has been rising tremendously, and those conformant to the programs reach 89% for Tantalum, 88% for tin, 87% for tungsten and 67% for gold (Young et al. 2019, p. 7). Those are known as chokepoints with good visibility. However, below these chokepoints, visibility gets blurred. Blockchain may help to establish the full visibility in the supply chain and satisfy consumer and regulatory expectations even for lower tiers in the supply chain.

All this information enables manufacturing and logistics enterprises a more preventive rather than reactive strategy. Outside the core business intelligence, many other data sources may be tapped to increase the knowledge of incidents not inherent in operational management.

## Conclusions

The growing number of price and supply risks in mineral raw material markets and the growing influx of data about these markets suggest that big data analytics and artificial intelligence could help to identify critical supply situations at an earlier stage. The fundamental starting point for any of those analyses is a good knowledge of relevant suppliers and supplier locations, ideally along the whole supply chain down to the mining and refining stages.

The mineral raw materials sector is—next to other natural resource sectors—the market segment, where “it all starts”. Accordingly, it is the starting point for tracking and analysing incidents, which transcend the whole value chain. Ranking the severity of incidents is an iterative process while collecting further experience with big data applications.

This paper shows that big data applications are a good approach to track risk incidents at an early stage, point to social and environmental risks in certain mines or mining districtions and possibly help to foresee critical developments and price impacts higher up the value chains. The adoption of big data analytics, especially in the short-term focus (nowcasting), is a novel approach and closes a gap in research in that important incidents, mostly unforeseen, may contribute to the estimation of supply interruptions or failures. The following conclusions can be derived out of this analysis:

1. A good database about the most relevant suppliers (by production size and/or product quality) in one market segment and their geographic locations is the essential input data. Unfortunately, databases covering value chains in different industry sectors are incomplete or not available, considering the pure size of information: more than 60 mineral raw materials at mining stage stand at the beginning of supply chains, more than 80 refined products are manufactured at an early metallurgical stage, followed by several 100 products of early intermediate stages and 1000 s of products at various intermediate stages further up the value chains, followed by 100,000 s semi-finished products using material of previous processing steps. Thus, the aim is to build up better knowledge about value chains in critical markets.
2. Web scraping, which counts the daily number of media posts at a specific supplier location, could be a useful tool to quickly identify media peaks. Media peaks only occur if the information is relevant to the market and the public and point to potential market disruptions. Good examples are the incidents at Las Bambas copper mine in Peru in connection with labour strikes blocking the supply of copper concentrates to global markets and at McArthur River zinc mine and several iron ore mines in Australia associated with disputes in connection with the expansion of mine production impacting indigenous sacred sites. While a good number of media posts are highly relevant to potential disruptions, many are not. A better combination of web scraping with artificial intelligence technology could improve the relevance of such media posts and thus refine the results.
3. Big data tools, which directly monitor incidents (incident monitoring) at specific supplier locations, are highly valuable to inform market participants about moderate, severe or extreme risks (Alerting system). However, these tools need to be adjusted further to the specific mineral raw material markets. Moderate or severe weather incidents like tropical storms are, for example, much more relevant for bulk commodities using trains or bulk terminals at harbours than other commodities which can be transported containerised using almost any harbour (e.g. iron ore, manganese ore mined and transported in Australia and Brazil). Other, more short-term severe incidents only had a negligible impact to supply chains, for example, a heavy earthquake which occurred near an important copper mine in Poland or severe fuel shortage in Venezuela potentially affecting iron ore mining. Ranking the severity of incidents in global markets is a major challenge in big data analytics. It is according to the authors the topic that needs the biggest amount of refinement. Fully automated tools suffer from noisy data that impair the analysis of the markets. Refining web scraping and incident monitoring tools using artificial intelligence in combination with in depth market intelligence will effectively contribute building more resilient supply chains.
4. Highly concentrated markets such as the lithium, cobalt or the platinum group metal (PGMs) markets may react more sensitive to distortions than less concentrated markets and thus need to be monitored more closely. For example, the two major producers of PGMs are South Africa and the Russian Federation. Severe incidents in both countries immediately would affect global supply or prices. Although not part of this analysis, a good example is the Russian invasion into Ukraine in 2022 and international sanctions against the Russian Federation, which caused price hikes for nickel and palladium and fears of supply disruptions. Moderate and severe incidents such as labour strikes and riots may also trouble the PGE market especially since they frequently occur in South Africa. Other incidents could also disrupt markets, especially when it is not known how long these incidents or following interventions last, for example, (i) potential disruptions by an observed mine worker accident in Zimbabwe or security measures and road blockages by government authorities in Zimbabwe, after a national politician was suspected to have been poisoned, (ii) the fuel spill at Nornickel’s Ni-Cu-PGE mine Norilsk followed by legal disputes or (iii) a production halt at a PGE smelter in South Africa.
5. Even in less concentrated markets like the copper market, incidents which may affect only a few percent of global production such as at Escondida mine in Chile (6.9% of global copper production) or Las Bambas mine in Peru (1.9% of global production) could have an effect on price or specific supply chains. For instance, the road blockage and strike at Las Bambas mine in Peru in combination with other simultaneous incidents caused excitement among market participants and increased the copper price.
6. The global COVID-19 pandemic, which initially impacted mineral raw material markets in many ways like disruption of transport logistics or production stops, did not had a lasting effect. Initially, the markets were highly at risk. However, most governments exempted critical industry from long-term lockdowns such as the mining and refining industry, for example, in Chile or South Africa, which eased the situation. Thus, only a minor share of global mine production really was affected. It therefore is important to follow up incidents and to observe incidents how they develop over time.
7. For this analysis, only the largest mines were selected for tracking. However, country-wide incidents such as new government regulations leading to environmental inspections and site closures in China could also affect a larger number of small and medium size mining companies, which together may have a high relevance to a mineral raw material market. This definitely is the case for China with a huge number of small- to medium-size companies, for example, in the zinc market, where all zinc producers together have a high share of global production. Another good example are announcements in Peru to increase royalties in the mining sector, which irritated copper miners: The presidential candidate of Peru had proposed to raise royalties on mineral sales and announced tax renegotiation plans, which increased the risk exposure for mining companies and troubled the copper market. Thus, severe country wide incidents may need to be ranked differently in big data analytics.
8. Country or regional specific risk indicators covering natural disaster, political violence, sustainability or environmental risk are useful to identify the best-performing socio-political frameworks in which mines operate. Although country indicators may not necessarily reflect the situation at a specific mine location, it is a first indication to look at the performance of suppliers in more detail.
9. The more mineral raw material markets are taken into consideration for such big data analytics, and the more they are continuously monitored, the better they are understood and can be compared with one another. This procedure may additionally benefit the discussion and decision process to favour sustainably produced mineral raw materials and possibly substitute one supplier by another.

There certainly is a lot more to learn about big data analytics applied to the mineral raw material markets. This relatively young science will help to identify risks faster, predict disruptions along value chains at an early stage and to make faster decisions in industry. Further experience will improve the learning curve using such analytical tools, help to identify relevant risks and help to refine the ranking of risks.

## Supplementary Information

Below is the link to the electronic supplementary material.

Supplementary file1 (XLS 48 KB)
